# Bipedalism or bipedalisms: The os coxae of StW 573

**DOI:** 10.1111/joa.14106

**Published:** 2024-07-22

**Authors:** Robin Crompton, Sarah Elton, Jason Heaton, Travis Pickering, Kristian Carlson, Tea Jashashvili, Amelie Beaudet, Laurent Bruxelles, Kathleen Kuman, Susannah K. Thorpe, Eishi Hirasaki, Christopher Scott, William Sellers, Todd Pataky, Ronald Clarke, Juliet McClymont

**Affiliations:** ^1^ Department of Musculoskeletal and Ageing Science, Institute of Life Course & Medical Sciences, Faculty of Health & Life Sciences, the W.H. Duncan Building University of Liverpool Liverpool UK; ^2^ School of Archaeology, Classics and Egyptology University of Liverpool Liverpool UK; ^3^ Department of Anthropology, Dawson Building Durham University Durham UK; ^4^ Department of Biology University of Alabama at Birmingham Birmingham Alabama USA; ^5^ Evolutionary Studies Institute University of the Witwatersrand Johannesburg South Africa; ^6^ Department of Anthropology University of Wisconsin Madison Madison Wisconsin USA; ^7^ Department of Integrative Anatomical Sciences, Keck School of Medicine University of Southern California Los Angeles California USA; ^8^ Department of Geology and Palaeontology Georgian National Museum Tbilisi Georgia; ^9^ Department of Archaeology University of Cambridge Cambridge UK; ^10^ TRACES, UMR 5608 CNRS Jean Jaurès University Toulouse France; ^11^ French National Institute for Preventive Archaeological Research (INRAP) Nîmes France; ^12^ School of Geography, Archaeology and Environmental Studies University of the Witwatersrand Johannesburg South Africa; ^13^ School of Biosciences University of Birmingham Birmingham UK; ^14^ Center for the Evolutionary Origins of Human Behavior University of Kyoto Kyoto Japan; ^15^ Department of Earth and Environmental Sciences University of Manchester Manchester UK; ^16^ Department of Human and Health Sciences Kyoto University Kyoto Japan

**Keywords:** *Australopithecus*, biomechanics, innominate, locomotion, neurobiological degeneracy, os coxae

## Abstract

There has been a long debate about the possibility of multiple contemporaneous species of *Australopithecus* in both eastern and southern Africa, potentially exhibiting different forms of bipedal locomotion. Here, we describe the previously unreported morphology of the os coxae in the 3.67 Ma *Australopithecus prometheus* StW 573 from Sterkfontein Member 2, comparing it with variation in ossa coxae in living humans and apes as well as other Plio‐Pleistocene hominins. Statistical comparisons indicate that StW 573 and 431 resemble humans in their anteroposteriorly great iliac crest breadth compared with many other early australopiths, whereas *Homo ergaster* KNM WT 15000 surprisingly also has a relatively anterioposteriorly short iliac crest. StW 573 and StW 431 appear to resemble humans in having a long ischium compared with Sts 14 and KNM WT 15000. A Quadratic Discriminant Function Analysis of morphology compared with other Plio‐Pleistocene hominins and a dataset of modern humans and hominoids shows that, while Lovejoy's heuristic model of the *Ardipithecus ramidus* os coxae falls with *Pongo* or in an indeterminate group, StW 573 and StW 431 from Sterkfontein Member 4 are consistently classified together with modern humans. Although clearly exhibiting the classic “basin shaped” bipedal pelvis, Sts 14 (also from Sterkfontein), AL 288‐1 *Australopithecus afarensis*, MH2 *Australopithecus sediba* and KNM‐WT 15000 occupy a position more peripheral to modern humans, and in some analyses are assigned to an indeterminate outlying group. Our findings strongly support the existence of two species of *Australopithecus* at Sterkfontein and the variation we observe in os coxae morphology in early hominins is also likely to reflect multiple forms of bipedality.

## Graphical abstract

There has been a long debate about the possibility of multiple contemporaneous species of *Australopithecus* in both eastern and southern Africa, potentially exhibiting different forms of bipedal locomotion. Here, we describe the previously unreported morphology of the os coxae in the 3.67 Ma *Australopithecus prometheus* StW 573 from Sterkfontein Member 2 (left), comparing it with variation in ossa coxae in living humans and apes as well as other Plio‐Pleistocene hominins. Statistical comparisons indicate that StW 573 and StW 431 (right) resemble humans in their anteroposteriorly great iliac crest breadth compared with many other early australopiths, with *Homo ergaster* KNM WT 15000 surprisingly having a relatively anterioposteriorly short iliac crest. StW 573 and StW 431 also appear to resemble humans in having a long ischium compared with Sts 14 and KNM WT 15000. A quadratic discriminant function analysis of morphology compared with other Plio‐Pleistocene hominins and a dataset of modern humans and hominoids shows that, while Lovejoy's heuristic model of the *Ardipithecus ramidus* os coxae falls with Pongo or in an indeterminate group, StW 573 and StW 431 from Sterkfontein Member 4 are consistently classified together with modern humans. Although clearly exhibiting the classic “basin shaped” bipedal pelvis, Sts 14 (also from Sterkfontein), AL 288‐1 *Australopithecus afarensis*, MH2 *Australopithecus sediba* and KNM‐WT 15000 *Homo ergaster* occupy a position more peripheral to modern humans, and in some analyses are assigned to an indeterminate outlying group. Our findings strongly support the existence of two species of Australopithecus at Sterkfontein and the variation we observe in os coxae morphology in early hominins is also likely to reflect multiple forms of bipedality.

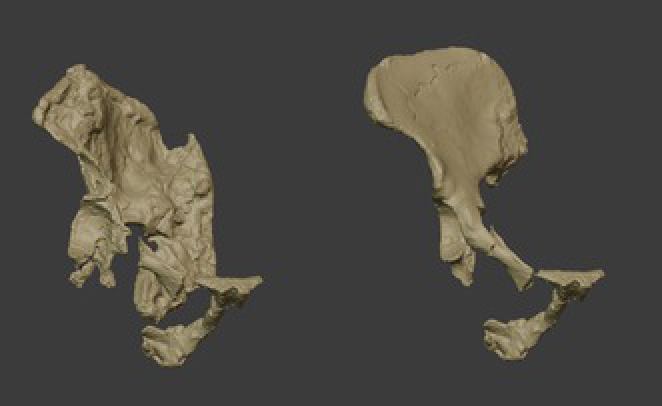

## 1 INTRODUCTION

**FIGURE 1:**
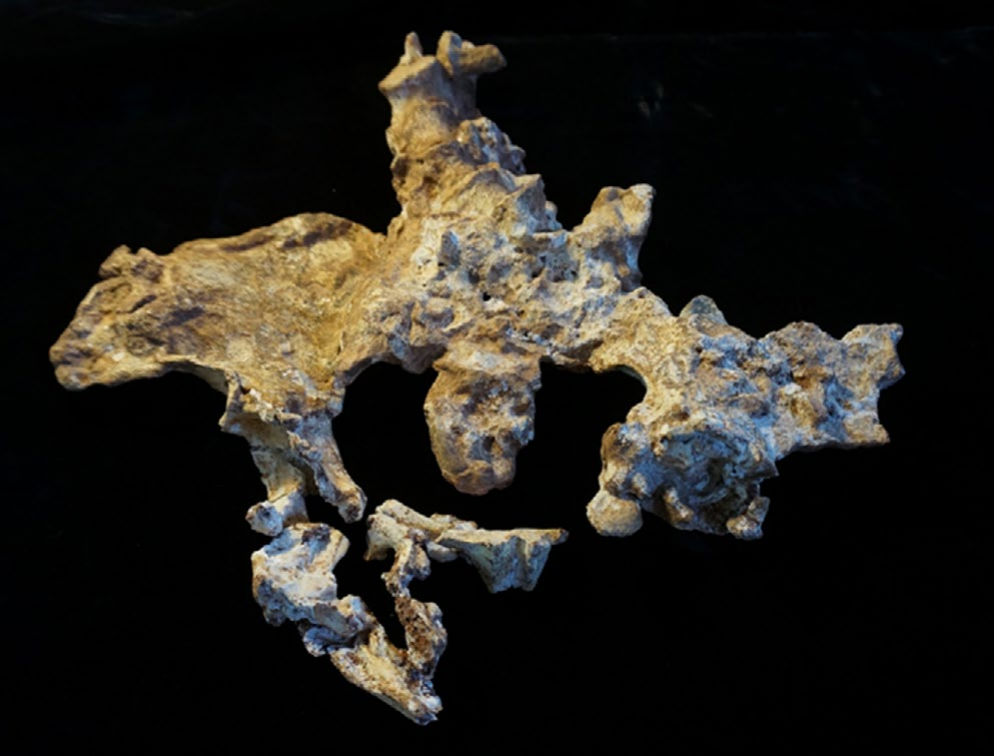
The original pelvis of StW 573 prior to restoration, ventral image. Note that in this image the ischiopubic ramus is as originally freed from matrix, prior to reconstruction, and it remains attached to other unassociated bony fragments. Not to scale.

**FIGURE 2:**
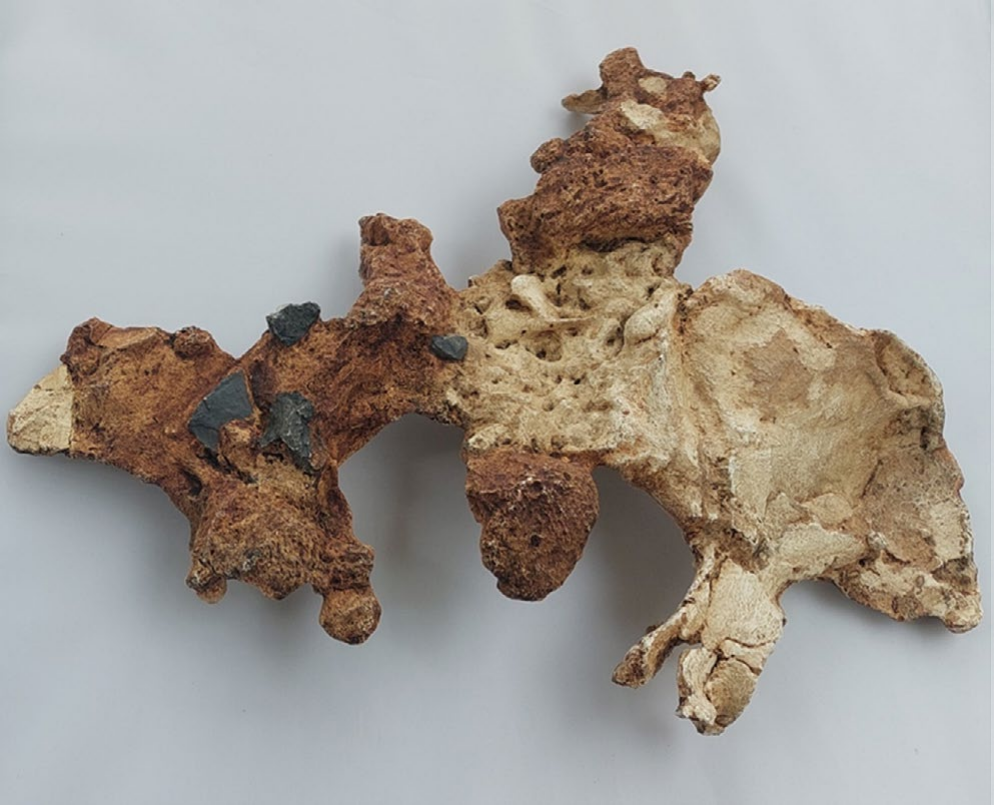
Dorsal aspect of a cast of the StW 573 pelvis. Not to scale.

**FIGURE 3:**
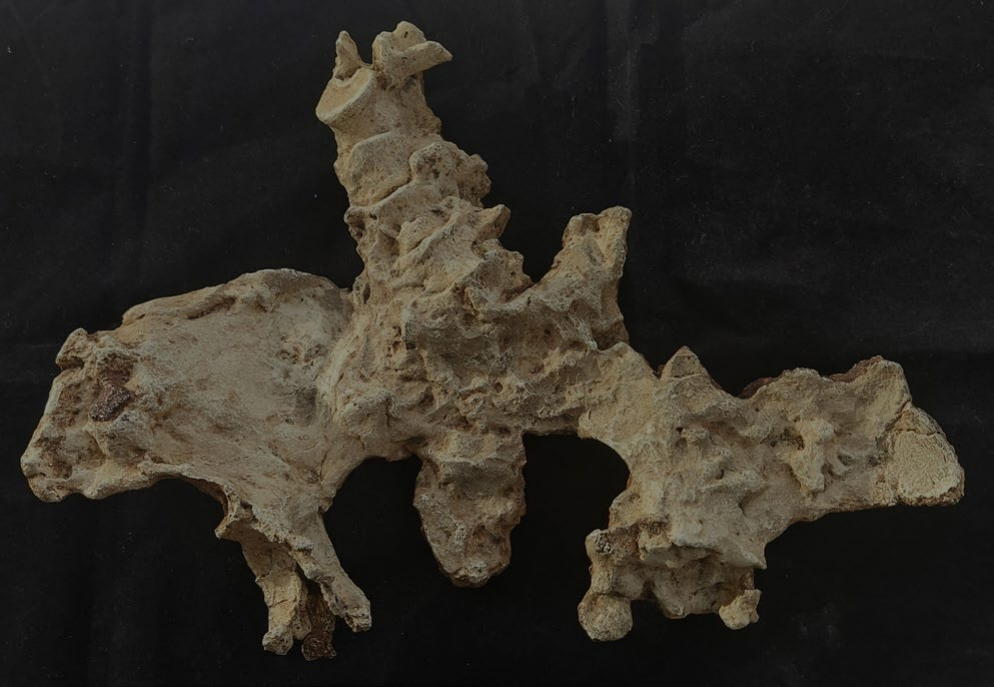
Ventral aspect of a cast of the StW 573 pelvis. Not to scale.

The StW 573 footbones were first reported by Clarke and Tobias (1995) as a partial foot, but later found to belong to a progressively more complete skeleton of *Australopithecus prometheus* from Member 2 at Sterkfontein. Discovered, excavated, and prepared by Clarke and team, it is now the most complete hominin individual prior to *Homo ergaster* KNM WT 15000. Elements of StW 573 have been published in a series of papers between 1998 and 2021 (Beaudet, Clarke, Bruxelles, et al., 2019; Beaudet, Clarke, De Jager, et al., 2019; Beaudet et al., 2020; Bruxelles et al., 2014, 2019; Carlson et al., 2021; Clarke & Tobias, 1995; Clarke, 1998, 1999, 2002,  2008, 2013; 2019a, 2019b, Clarke & Kuman, 2019; Crompton et al., 2022; Deloison, 2003, 2004; Heaton et al., 2019; Stratford & Crompton, 2021). Following Clarke's physical restoration, work is ongoing to virtually reconstruct crushed elements of the skeleton, including the sacrum and lumbar vertebrae; the scapula has already been reconstructed (Carlson et al., 2021). The present paper reports the unreconstructed os coxae (Figures 1, 2, 3): reconstructions are currently under way and will be published in due course.

StW 573, *Australopithecus prometheus*, comes from Member 2 of the karstic Sterkfontein Formation, near Johannesburg, South Africa (see Partridge et al., 2003 for a description of the six geological members). The completeness of the StW 573 skeleton results from an unusual combination of circumstances. The individual fell into a deep shaft, was mummified in dry conditions, and later calcified within a debris slope under wet conditions, thus preserving many bones in articulation. There is no evidence of predator or scavenger damage to any of the elements. Burial conditions and taphonomy are reported in Clarke (2019a, 2019b), and stratigraphy by Bruxelles et al. (2014, 2019). The age of StW 573 has been determined as ca. 3.67 million years (Ma) (Granger et al., 2015, 2022).

The Sterkfontein caves provide a long stratigraphic sequence documenting the evolution of hominins, fauna, landscape, environmental change and early stone tool technology. Member 4, dating to ca. 3.4 Ma (Granger et al., 2022), is the world's richest single *Australopithecus*‐bearing deposit, containing two species of that genus, together with diverse and numerous faunal remains. Below Member 4 is the extensive, fossiliferous, and yet‐to‐be excavated Member 3, which overlies Member 2, which yielded the *Australopithecus* skeleton StW 573. Overlying Member 4 are the Member 5 infills which, through many years of excavation, have produced fossils of early *Homo*, *Paranthropus*, and evidence for nearly a million years of early stone tool technology evolution (Kuman, 1994a, 1994b; Kuman & Clarke, 2000; Kuman & Field, 2009). Sterkfontein Member 2 fauna indicates a palaeohabitat of rocky hills covered in brush and scrub, but valley bottoms with riverine forests, swamps and standing water (Pickering et al., 2004). The presence of ancient gravels preserved only 300 m from Sterkfontein (Pickering et al., 2004), indicate there was in the past a large, slow flowing river in the base of the valley. This, together with fossils in the younger Member 4 of a species of forest vine that today only occurs in central and western Africa (Bamford, 1999), suggests a remnant Miocene tropical forest in the valley bottom.

**FIGURE 4:**
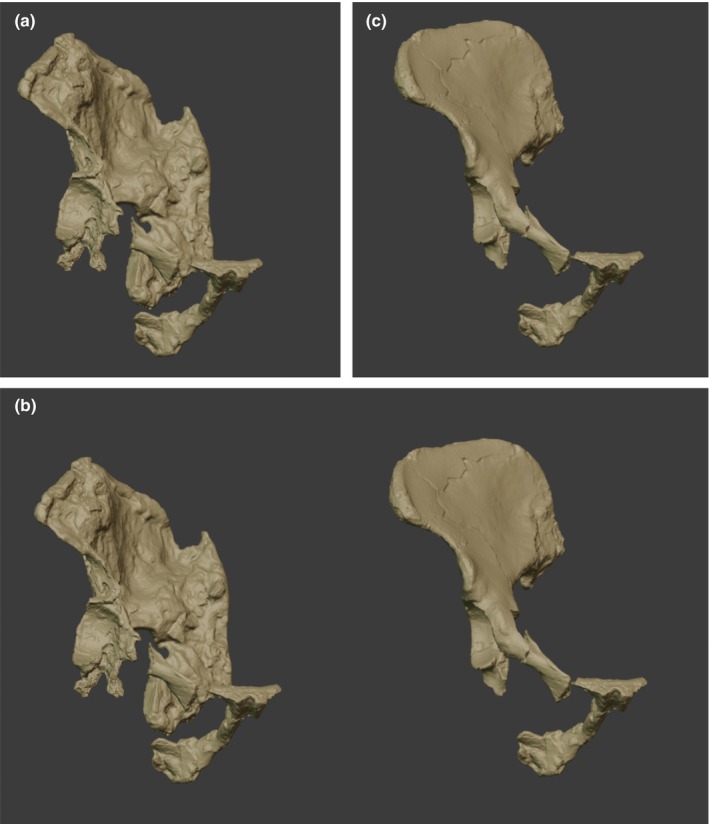
(a) A digital model of the right os coxa of StW573 with the StW 573 ischiopubic ramus inserted, and the StW431i iliopubic ramus. (b) The digital model of the right os coxa of StW573, compared with a digital model of the right os coxa of StW 431, both with the StW 573 ischiopubic ramus, and the StW431i iliopubic ramus inserted. (c) The digital model of the right os coxa of StW 431, with the StW 573 ischiopubic ramus, and the StW431i iliopubic ramus inserted.

**FIGURE 5:**
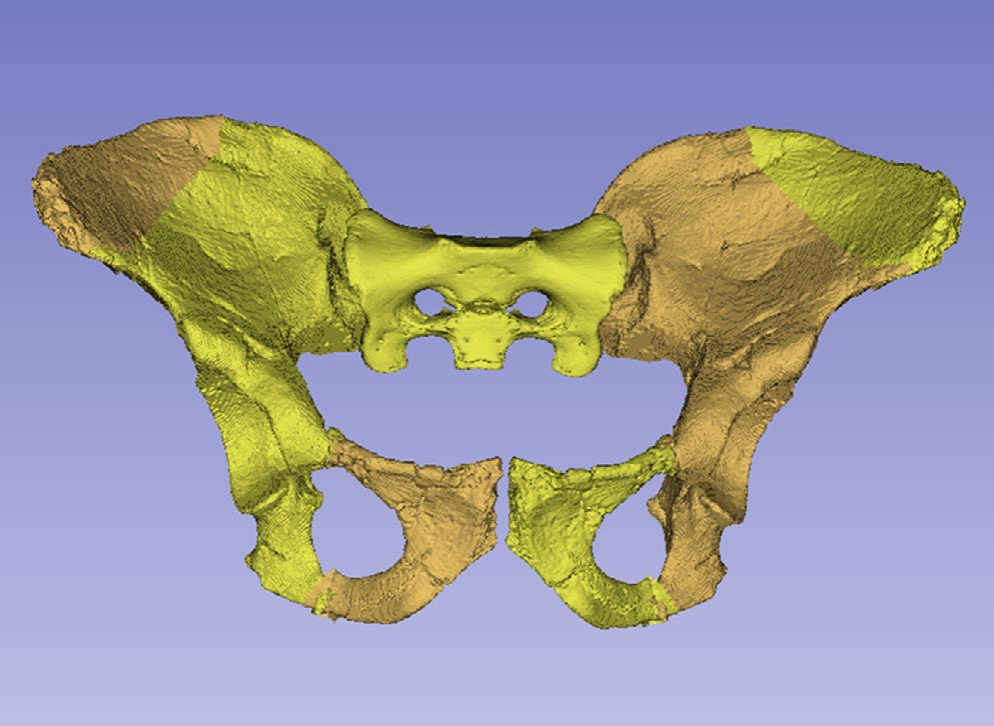
The Sts 14 pelvis, as reconstructed by Berge and Goularas (2010). Not to scale.

On the basis of craniodental evidence, Clarke (1998, 2008, 2013) has argued that there are two species of *Australopithecus* at Sterkfontein. This was further detailed by Clarke and Kuman (2019) in their diagnosis of the StW 573 skull as *Australopithecus prometheus*. Postcranial evidence also suggests the presence of more than one hominin species at Sterkfontein (e.g. Berge et al., 2007; Fornai et al., 2021), although an extensive 2019 review of postcranial material was ambivalent about such taxonomic heterogeneity being reflected in the ossa coxae of Sts 14 and StW 431 (Grine, 2019). Alongside StW 573, material assigned to Sts 14 and StW 431 has been pivotal to the debates over the Sterkfontein species question. Some of the debate centres around the accuracy of reconstructions of these fragmented fossils as summarised in greater detail in Grine (2019). In brief, there are morphological differences in the Haeusler (2002) StW 431 pelvic reconstruction compared with the Kibii and Clarke (2003) reconstruction. The latter (see Figure 4a,b), which we adopt in the present study, was informed by newly discovered fragments with indisputable contacts between the sacrum and the ilium, which were not known at the time of Haeusler (2002) reconstruction. It is also possible that a more recent reconstruction of Sts 14 (Figure 5), by Berge and Goularas (2010) – which we again use in the current study and which has more lateral flare in the iliac blades – has reduced the overall difference in morphology between Sts 14 and StW 431 previously reported by Grine (2019). Sexual dimorphism may also complicate interpretation (sensu Grine, 2019) but in their study of the sacrum, Fornai et al. (2021) investigated this possibility rigorously, concluding that the magnitude and pattern of shape differences between Sts 14 and StW 431 goes beyond what could be explained by shape dimorphism, allometry or age.

The pelvis, connecting the trunk and hindlimb at the core of the body, and providing attachment sites for the muscles of the abdomen, pelvic floor and back as well as the hip, may be expected to be highly informative about posture and locomotion, as well as, in females, obstetrics. The pelvis comprises the two ossa coxae, or hip bones, plus the sacrum and coccyx, but here, we focus only on the os coxae, commonly referred to as the innominate. The os coxae comprises the ilium, the ischium and the pubis. Bipedal locomotion must be assumed to have exerted a profound selection pressure on the os coxae: for example, bipedal walking demands that the body is supported on only one limb during its swing phase, requiring the small hip muscles to be capable of stabilising the trunk and this in turn resulting in a curved, mediolaterally orientated ilium (Aiello & Dean, 1990). The human ilium is also much reduced in height compared with those in great apes, helping to offset load bearing stress via the sacroiliac joint (Aiello & Dean, 1990). The result of these and other adaptations is the “basin‐shaped” bipedal pelvis that is so diagnostic of bipedality, alongside profound functional changes to the hip and thus its muscles, such as the hamstrings and iliopsoas, influencing the morphology of the ischium and pubis respectively (Aiello & Dean, 1990). Unsurprisingly, the hominin pelvis has been subject to considerable research.

**FIGURE 6:**
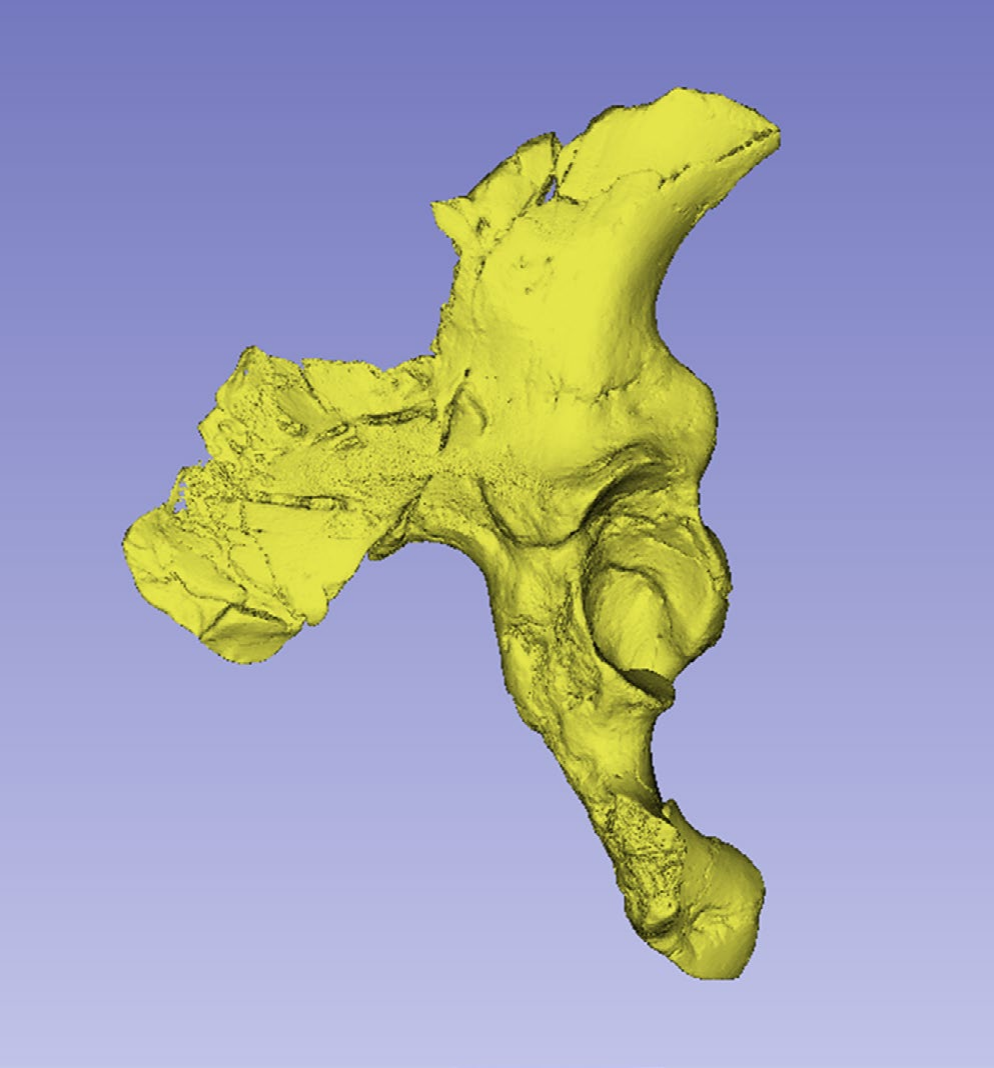
Os coxae of SK 50 prior to reconstruction of upper iliac blade and crest by Berge (1991). Not to scale, actual measurements: ***a*** = 60.86, ***b*** = 168.12, ***c*** = 99.008, ***d*** = 122.05, ***e*** = 44.976, ***f*** = 25.72, ***h*** = 38.6, ***j*** = missing, ***l*** = missing, ***m*** = 122.85, ***n*** = 58.606, ***o*** = 67.8.

For example, Napier (1964) comments in particular on the morphological distinctions which then appeared to characterise the ‘gracile’ australopiths, genus *Australopithecus* versus the ‘robust’ australopiths, genus *Paranthropus*. He notes a marked size difference between *Paranthropus* SK 50 (Figure 6) and *Australopithecus* Sts 14, and expresses the view that an iliac pillar and buttress are lacking in both, suggesting that this implies functional distinctions. He remarks that *Paranthropus* resembles modern *Homo* in a rather projecting anterior inferior iliac spine but differs in a less prominent anterior superior iliac spine. He further states that the ischium is considerably longer in *Paranthropus* than in modern *Homo*. He notes in particular the absence of an iliac pillar in *Paranthropus* as suggestive that *Paranthropus* lacked the ability to transfer weight from one leg to the other during bipedal walking. Robinson's extensive work (e.g. 1972, from whence we draw most of our variables) arrives at a similar conclusion, adding that *Paranthropus*, but not *Australopithecus africanus* had ‘incomplete emancipation from the trees’ (p. 252). However, McHenry (1975) found that the functional length of the ischium (from the centre of the acetabulum to the distalmost point on the ischial tuberosity, and therefore most likely the hamstring mechanism as a whole) was very similar in *Paranthropus robustus* and *Australopithecus africanus*, taking into account the small size of Sts 14. He concluded that there was little functional difference in gait between the South African hominins as a whole and modern *Homo sapiens*. More recently, Haeusler (2002) reported in detail on the functional morphology of hip musculature in Sts 14 in comparison with AL 288‐1 *Australopithecus afarensis*, making extensive use of the particularly clear muscle markings on the ilium of StW 431, from Bed 4 at Sterkfontein. He pointed out that the gluteus maximus was likely equally large as it is in modern humans and similarly originated directly from the ilium, in contrast to the case in nonhuman great apes. He argued that the pelves of Sts 14 and StW 431 resemble each other more closely than either does AL 288‐1. Most recently, based on the technique of Brassey et al. (2018), Wiseman (2023) used volumetric muscle modelling, guided by imaging as well as muscle marking, to reconstruct 36 muscles of the pelvis and lower limb, and their potential interaction. The study confirms that AL 288‐1 was able to walk upright, but shows it could also engage in a wider repertoire of behaviours similar to those seen in bonobos.

This notwithstanding, there has been surprisingly little work that considers the os coxae in an ecomorphological context, particularly whether its functional morphology indicates that multiple forms of bipedality existed. Arguments that multiple forms of bipedality and multiple species existed within *Australopithecus* in eastern Africa have been based primarily on footprint shape. Re‐excavation and reanalysis of the Laetoli footprints has provided data that McNutt et al. (2021) claim show significant differences between the five Laetoli A footprints and those at Laetoli G, and hence multiple hominin species at Laetoli. However, the original preservation of the footprints was poor (Leakey, 1987; Tuttle, 1987) and the sample size remains small, so circumspect interpretation is required (Bates, Collins, et al., 2013 and Bates, Savage, et al., 2013, McClymont et al., 2016, McClymont et al., 2021). A recent analysis of the Laetoli G footprint trail, contemporaneous with StW 573 (McClymont & Crompton, 2021), revealed that only under very small areas of the foot can external function be statistically distinguished from those made by modern humans. Statistically reliable assessments of an individual's foot pressure characteristics require a minimum of 100 step records in laboratory conditions, based on studies of healthy, modern Western humans (McClymont et al., 2021). The Laetoli G trails, which remain the longest continuous record of footfall characteristics of any australopith, provide no more than nine reliable step records. There is, additionally, no direct equivalency between foot‐pressure (and hence foot function) and foot‐prints (Bates, Savage, et al., 2013) so considerable caution must be applied when inferring the former from the latter.

Comparison of the partial feet from Dikika (3.32 Ma) and Woranso‐Mille (3.4 Ma) indicates that the presence of an opposable hallux in the Burtele foot (BRT‐VP‐2/73) from Woranso‐Mille makes it more similar to *Ardipithecus ramidus* than it is to the Dikika and other *Australopithecus afarensis* feet (Haile‐Selassie et al., 2012). Thus, according to Haile‐Selassie et al. (2012), the Burtele hominin may not have had a human‐like “toe off” during gait, which might imply the presence of multiple hominin species. However, observations of a modern bonobo walking bipedally published more recently show clear hallucal toe‐off (Bates, Collins, et al., 2013; also see Vereecke et al., 2003 and d'Août et al., 2004). This demonstrates that at least one non‐human ape can exert toe‐off forces with the hallux, highlighting the complexities of drawing functional conclusions from fossil specimens. Plasticity in foot function irrespective of bony morphology is also evident in modern humans, some of whom can adduct the abducted hallux powerfully enough to vertically climb small vines (Venkataraman, Kraft, & Dominy, 2013).

One interpretation of the StW 57013 foot is that it “lacked the ape‐like ability to oppose the great toe” (McHenry & Jones, 2006: 534), although its reliance on arboreality is clear in other parts of its skeleton (Crompton et al., 2022). This, alongside the relatively recent observations about human foot functional plasticity (Venkataraman, Kraft, & Dominy, 2013) again underlines the challenges in inferring behaviour from pedal morphology. In their extensive review, Behling et al. (2023: page 1) point out that:

Based on current evidence, it is clear that the mechanical function of the foot is highly versatile. This function is adaptively controlled by the central nervous system to allow the foot to meet the wide variety of demands necessary for human locomotion. Importantly, it seems that substantial joint mobility is essential for this function.

Bernstein (1967) observed that the degrees of freedom (DOFs) in limb joint movement, especially in the hands and feet, are far higher than theoretically required for any given movement. These ideas on redundancy have been elaborated and extended, with Wainwright (1991) arguing that they lie at the heart of our understanding of adaptation and ecomorphology. More recently, Bernstein's ideas have been expanded (e.g. Edelman & Gally, 2001; Latash et al., 2002) into the concept of neurobiological degeneracy, “the ability of elements that are structurally different to perform the same function or yield the same output” (Edelman & Gally, 2001, p. 13763). The importance of degeneracy to ecomorphology further lies in providing a source of variability both for exploration during learning and for adaptation to changing environment (sensu Komar et al., 2015).

How is this relevant to our study of the StW 573 os coxae? The more bones, ligaments, and tendons there are in any given region, and the more joints that exist between them, the more ways a given motion, and thus a given external function, can be performed (see e.g. McClymont and Crompton, 2021; McClymont et al., 2022): distal joint systems can and do exert similar external effects in many different ways. If even proximal joints between individual bones can require up to 6 DOFS (Manafzadeh & Gatesy, 2021), reducing the predictability of their motion, then that must be the case distally for each of the many joints in the hand and foot, so that predictability of their conjoined interactions with the external world must be very low. Thus, given the complex structure of the foot and hand, consisting of numerous bones, muscles, and ligaments, a deeper understanding of locomotor variation and adaptation in early hominins may be better served by studying fossil evidence of proximal joint systems, such as the os coxae. These joint systems exhibit many fewer joints than the hands and feet, and thus provide a potentially more secure perspective on locomotion and inferences about variation in behaviour and thus for taxonomy.

Our study provides a description and statistical comparison of the right ilium of StW 573 as excavated and prepared by Clarke, with brief notes on the morphology of the reconstructed ischiopubic ramus. We evaluate similarities and differences of ossa coxae morphology among several Plio‐Pleistocene hominins, to investigate whether there are affinities between StW 573 and StW 431 as conspecifics in *Australopithecus prometheus* to the exclusion of Sts 14, *Australopithecus africanus*. We evaluate whether pelvic morphology implies the presence of multiple bipedal modes, and place our findings within the theoretical framework of degeneracy.

## 2 MATERIALS AND METHODS

For this study, we measured 3D digital models of ossa coxae derived from surface scans, using 3D Slicer 5.2.2 software. Rationale for subject selection is given in Appendix S1.

There is a considerable literature on the accuracy of measurements made by callipers on originals versus those made by electronic methods. Although digital measurements made on CT models appear to be the most accurate (see e.g. Abraham et al., 2021), measurements on models made from surface scans are the next most accurate and further crucially, the use of digital models avoids damage from callipers or styli to irreplaceable specimens. The left ilium of StW 573 is too badly crushed (see e.g. Figures 2 and 3) to be reconstructed physically, so for measurement it was replaced with the mirrored right ilium.

**FIGURE 7:**
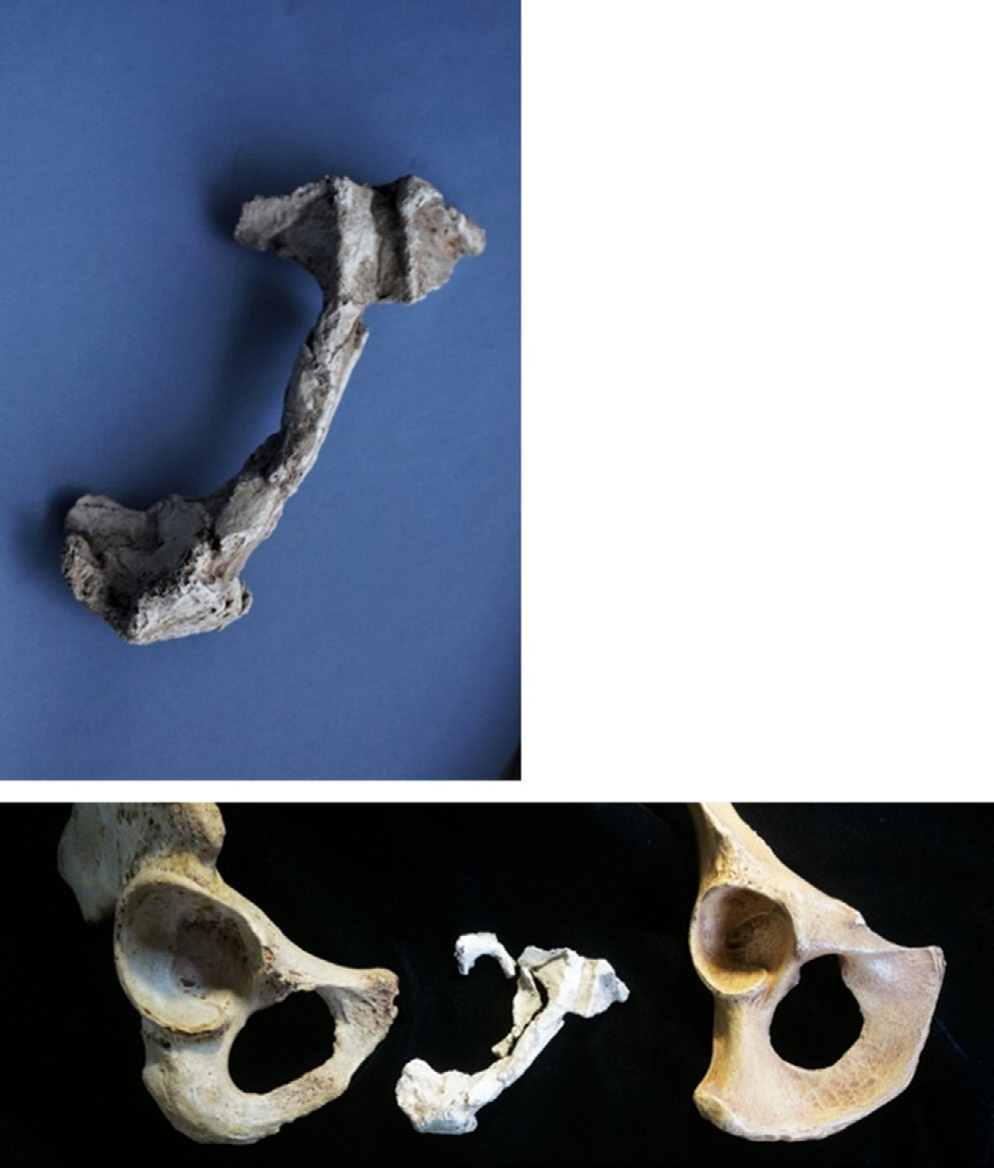
The StW573 ischiopubic ramus after reconstruction, and (below centre) contrasted with that of an human (below left) and a chimpanzee (below right). Note in the lower centre image our inclusion of the fragments of the margins of the obturator foramen, which unfortunately do not make good contacts and have not, therefore been included with the rest of the ischiopubic ramus elsewhere.

**FIGURE 8:**
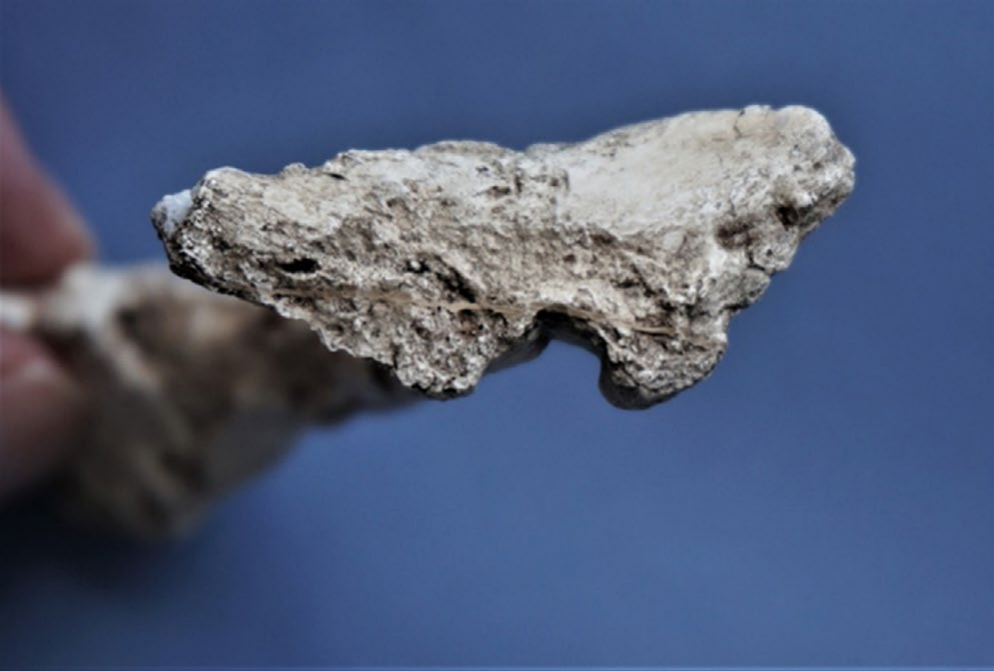
Dorsal perspective of the original StW 573 symphyseal fragment before reconstruction, to show U ‐shaped ventral groove.

**FIGURE 9:**
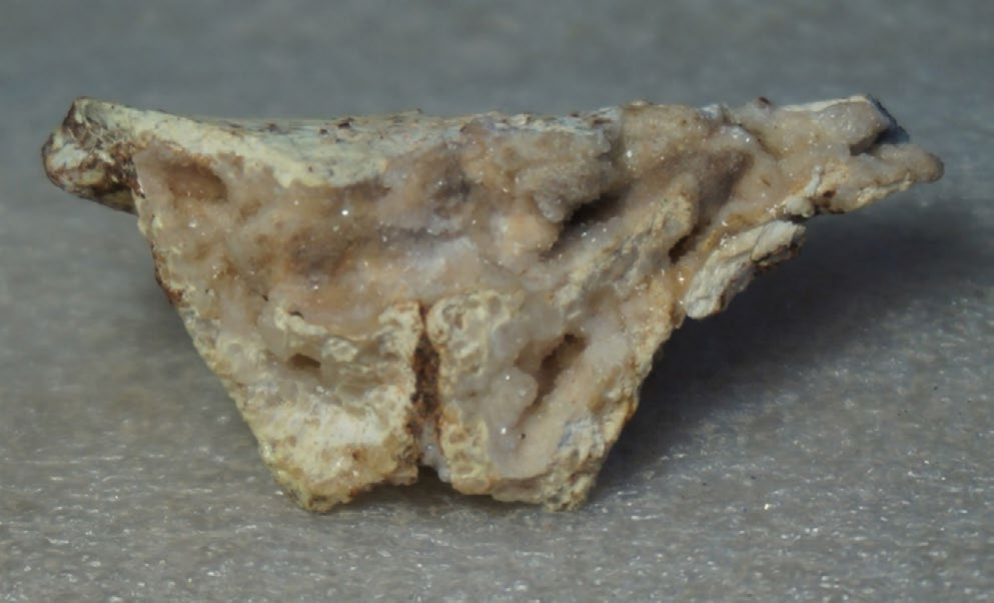
The StW 573 pubic symphysis before reassembly, dorsal view, to show symphysis and crystalline formations.

The ischiopubic ramus as prepared and physically reconstructed by Clarke (Figures 7, 8, 9) was surface‐scanned and inserted in its probable position in the digital model, before measurement (see Figure 4a). The StW 573 ischiopubic ramus was also similarly inserted into the model of StW 431, as it is a close size match, to give an overall impression of the complete pelvis (see Figure 4b,c). In both cases, we inserted a mirrored StW 431i iliopubic ramus (see Berge et al., 2007; Haeusler & Ruff, 2020) to enhance positioning. In neither case, should this be taken as a proposed reconstruction: Berge et al. (2007) and Haeusler and Ruff (2020) should be consulted for this.

**FIGURE 10:**
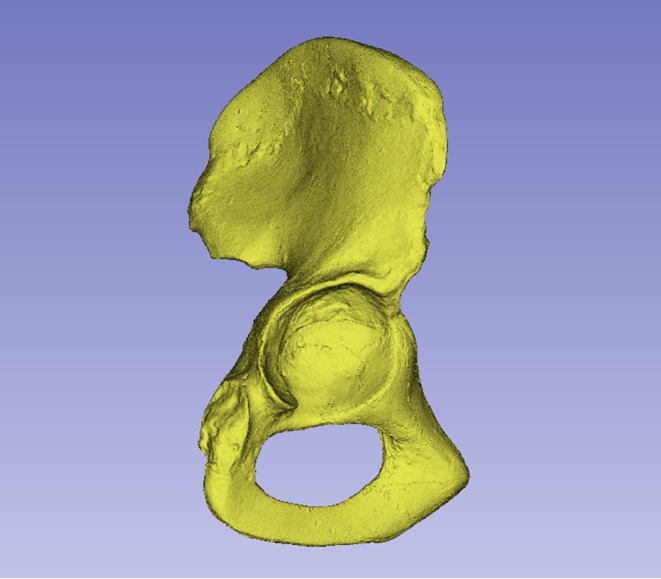
Heuristic model of *Ardipithecus ramidus* pelvis, lateral view. Courtesy of C. Owen Lovejoy. Not to scale.

**FIGURE 11:**
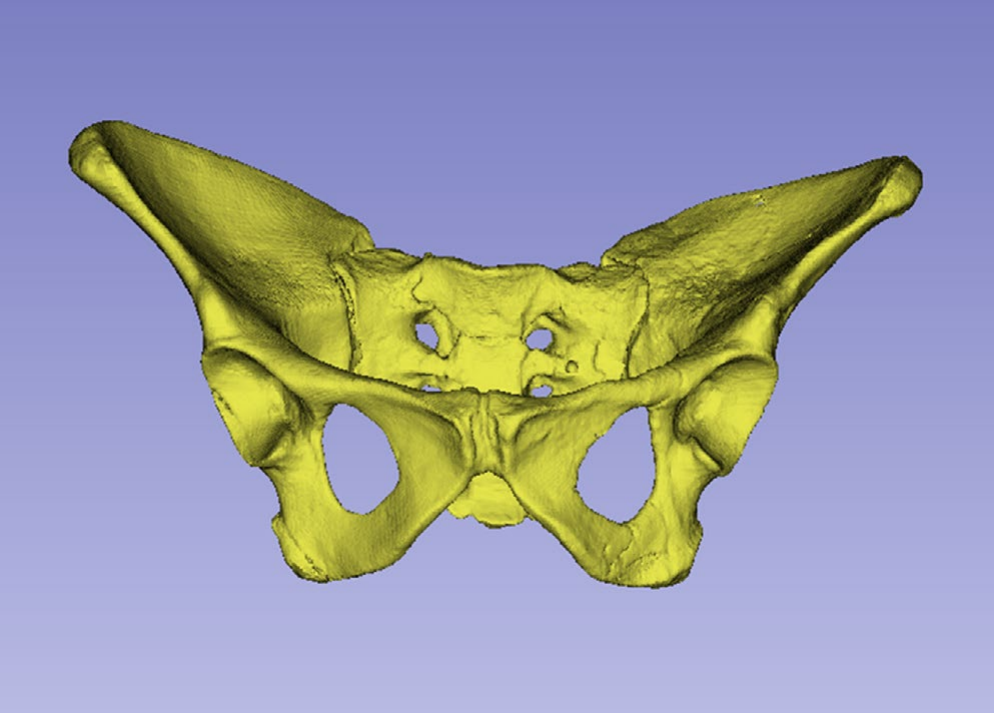
Digital model of pelvis of AL 288–1 courtesy of C. Owen Lovejoy. Not to scale.

**FIGURE 12:**
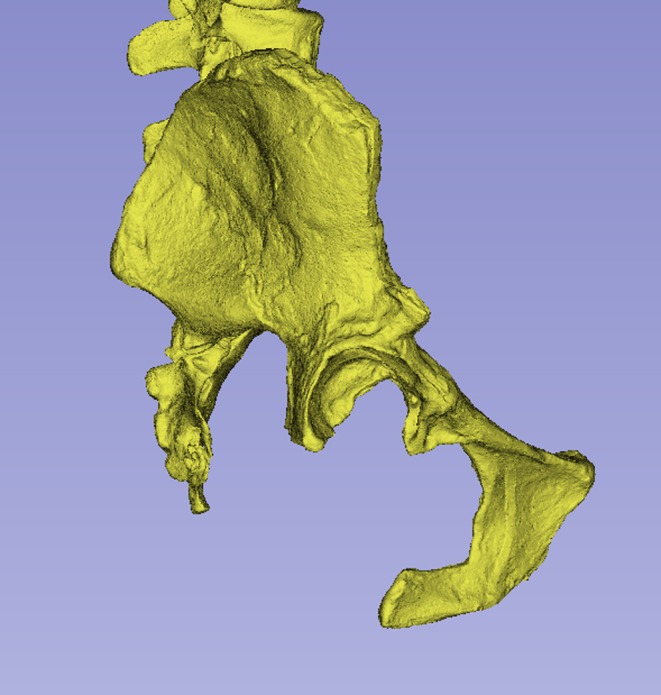
MH2 *Australopithecus sediba* os coxae/innominate (with lower spine and sacrum). Kibii et al. (2011) reconstruction. Not to scale.

**FIGURE 13:**
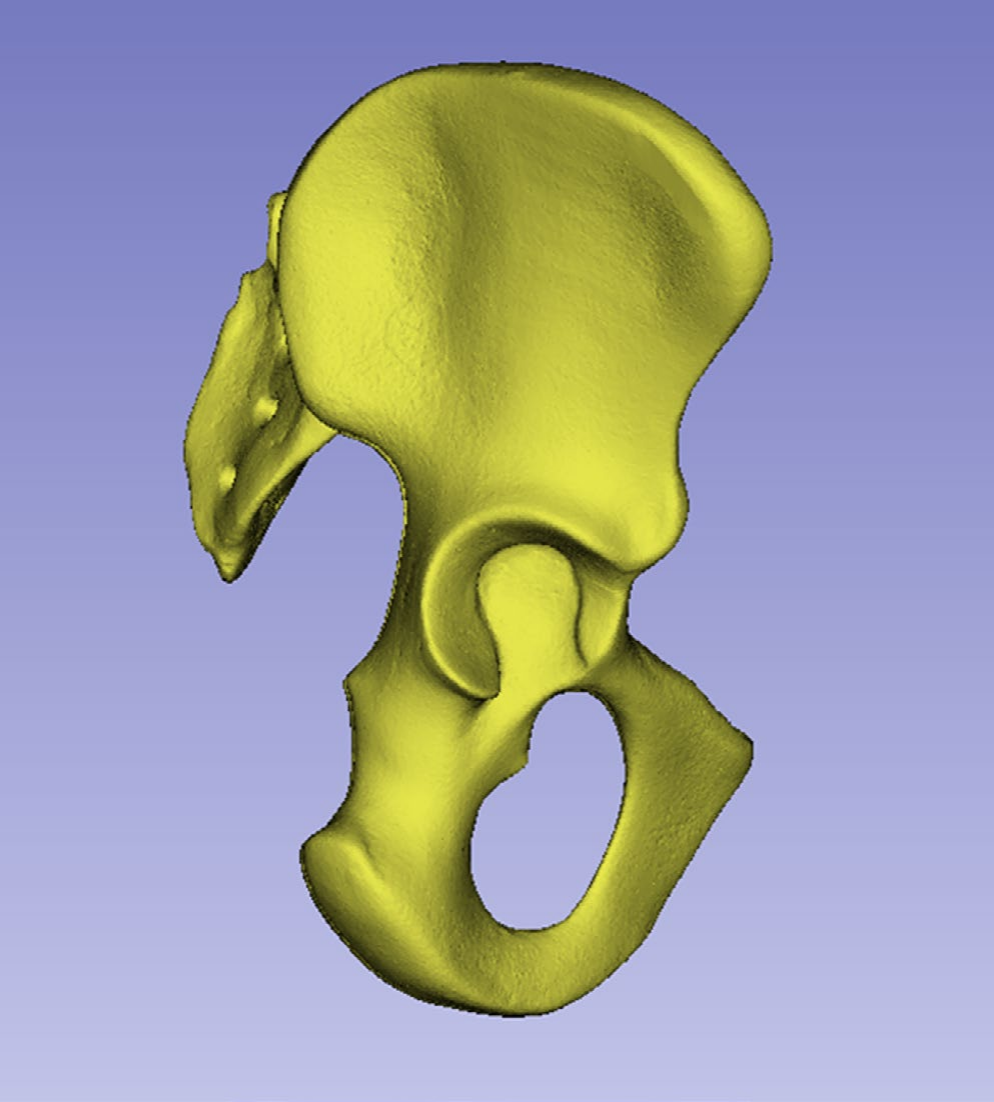
KNM‐WT 15000 right os coxae, Walker and Ruff (1993) reconstruction. Not to scale.

The measurement set (Table 1) followed that used by Robinson (1972) except that ***l*** was measured to the centre of the acetabulum, as Robinson (1972) himself said was preferable, and we added ***imb***, minimum breadth of the ilium, from Berge and Kazmierczak (1986). Humans and great apes are the most meaningful extant comparators for our study but we include the lesser apes as an outgroup. Within humans and great apes, there is considerable intra‐ and interspecific size difference but we take an ecomorphological approach and do not size‐adjust data, on the basis that within such a closely‐related group of animals, size acts as an ecological driver and hence provides important evolutionary information. Thus, the comparative sample comprised 155 modern hominoid specimens alongside ossa coxae from seven Plio‐Pleistocene hominins (Table 2 and Figures 1, 2, 3, 4, 5, 10, 11, 12, 13). All measurements on the 3D digital models were made by Crompton using a Wacom ET 0405A‐U tablet and Wacom EC 120‐OK mouse.

**TABLE 1:** Key to measurements.

***a***	Posterior superior iliac spine (psis) to caudalmost point on auricular surface
***b***	Anterior superior iliac spine (asis) to psis
***c***	Asis to cranialmost point on acetabulum
***d***	Asis to caudalmost point on auricular surface
***e***	Caudalmost point on auricular surface to ischial spine
***f***	Ischial spine to cranialmost point on ischium
***h***	Maximum diameter of acetabulum
***j***	Cranialmost point on pubis to caudalmost point on pubis
***l***	Centre of acetabulum to cranialmost point on pubis
***m***	Centre of acetabulum to cranialmost point on iliac crest
***n***	Caudalmost point on auricular surface to cranialmost point on acetabulum
***o***	Centre of acetabulum to distalmost point on ischium
***imb***	Minimum breadth of ilium

**TABLE 2:** Comparative sample used in this study.

Taxon/specimen	*N*	Hominin locality	References, notes and figure numbers
Gorilla	19	–	
Human	59	–	
Gibbon	15	–	
Chimpanzee and bonobo	29	–	
Orangutan	20	–	
Siamang	13	–	
*Total modern sample*	155	–	*–*
ARA‐VP‐6/500, *Ardipithecus ramidus*	–	Aramis	Heuristic model, see Lovejoy et al. (2009), Figure 11
AL 288‐1, *Australopithecus afarensis*	*–*	Hadar	See Johanson et al. (1982), Tague and Lovejoy (1986), Figure 12
StW 573, *Australopithecus prometheus*	–	Sterkfontein Member 2	This paper, Figure 1
StW 431, *Australopithecus prometheus*		Sterkfontein Member 4	See Kibii and Clarke (2003), Figure 3
Sts 14, *Australopithecus africanus*	*–*	Sterkfontein Member 4	See Robinson (1972) and Haeusler (2002), but model used is as reconstructed by Berge and Goularas (2010). Figure 4
MH2, *Australopithecus sediba*	–	Malapa	Kibii et al. (2011) Figure 13
KNM‐WT 15000, *Homo ergaster*	–	West Turkana	Walker and Ruff (1993), Figure 14

**FIGURE 14:**
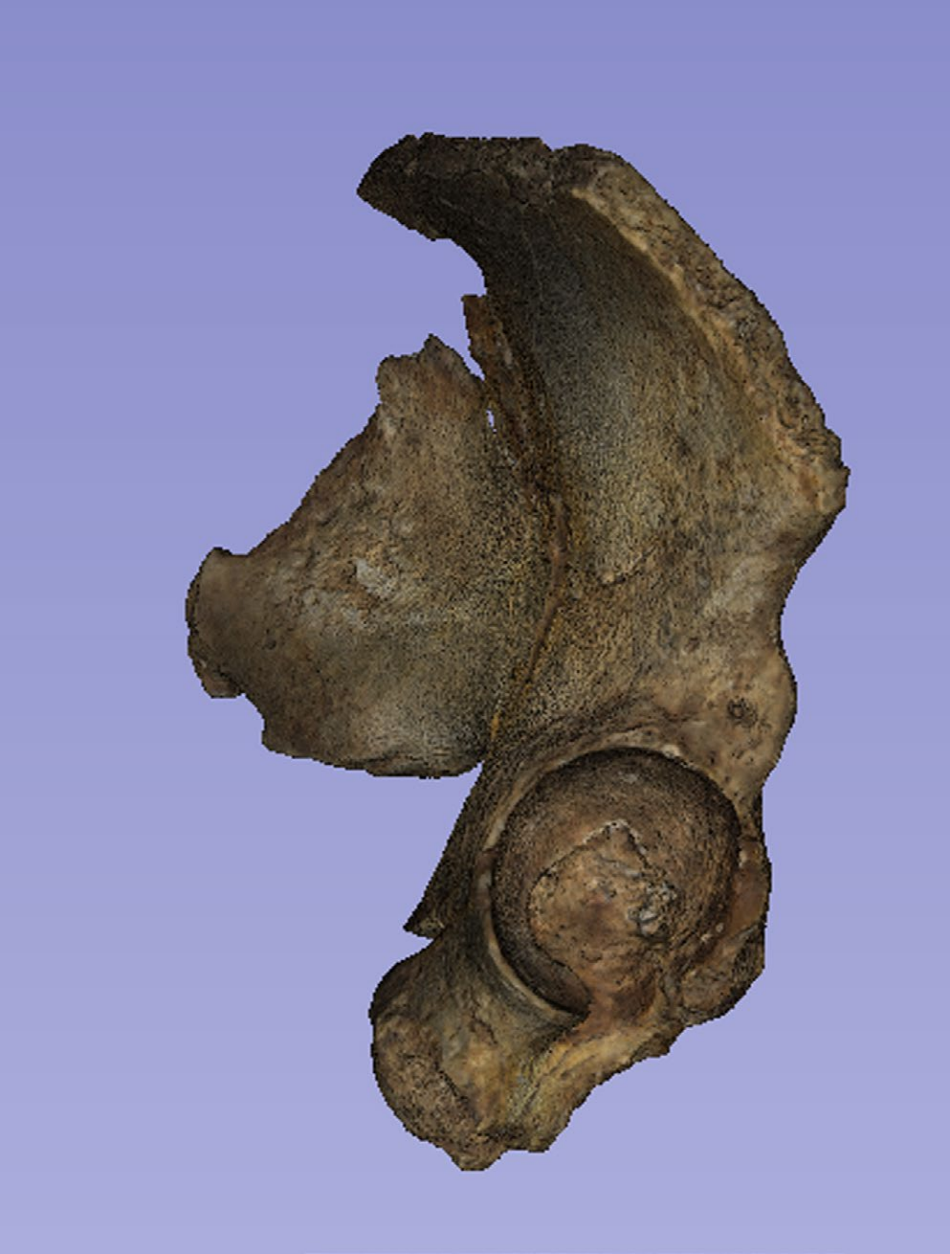
Os coxae of SK 3155 b (Robinson, 1972). Not to scale, actual measurements, to nearest mm: ***a*** = 70, ***b*** = 52, ***c*** = 53, ***d*** = 87, ***e*** = missing, ***f*** = missing, ***h*** = missing, ***j*** = missing, ***l*** = missing, ***m*** = 79, ***n*** = missing, ***o*** = missing, ***imb*** = missing.

**FIGURE 15:**
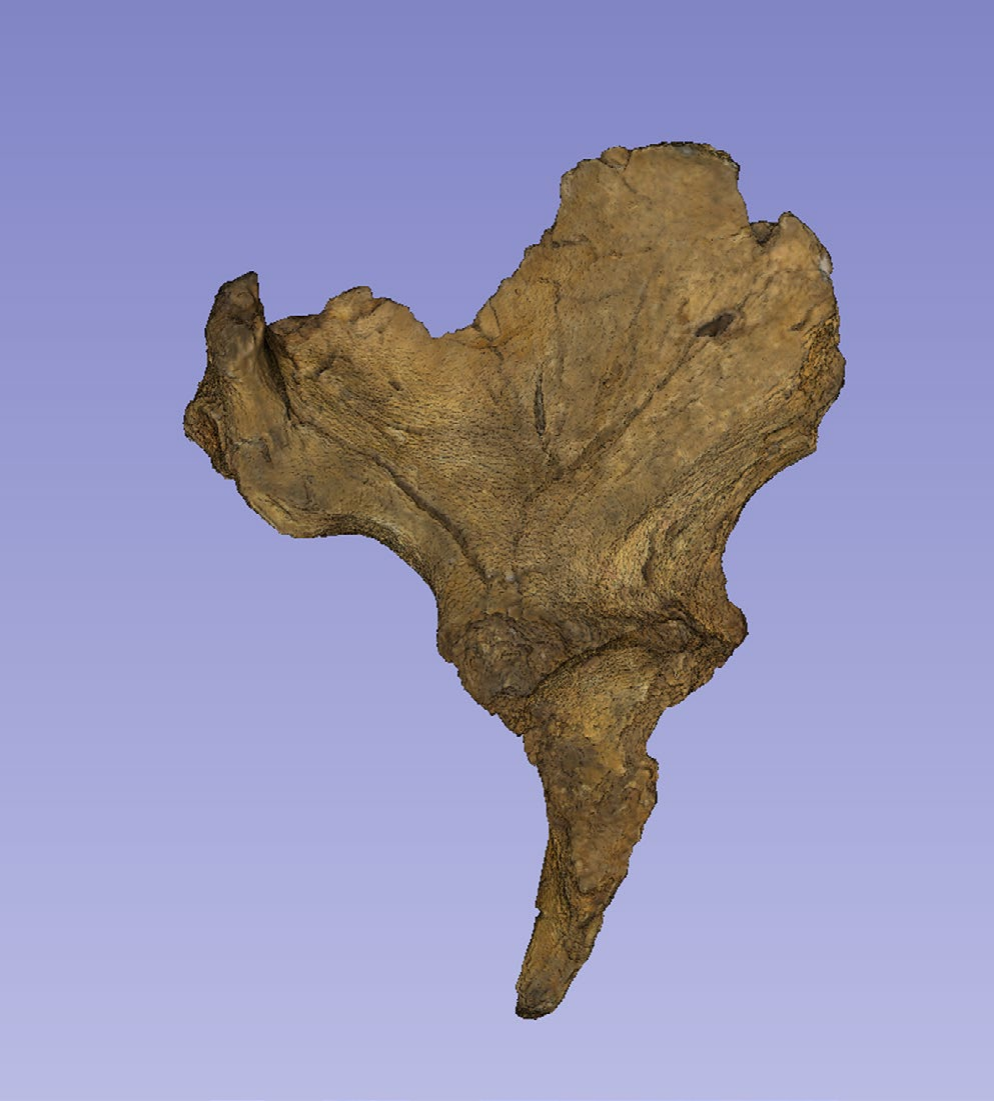
Os coxae of Sts 65. (Brain, Vrba and Robinson, 1974.) Not to scale, actual measurements, to nearest mm: ***a*** = 70, ***b*** = 52, ***c*** = 53, ***d*** = 87, ***e*** = missing, ***f*** = missing, ***h*** = missing, ***j*** = missing, ***l*** = missing, ***m*** = 79, ***n*** = missing, ***o*** = missing, ***imb*** = missing.

Other hominin os coxae exist but were too incomplete or distorted to include in the statistical element of this study. Those measurements which could be made are presented, for reference only, with the Figures for each. For SK 50 *Paranthropus robustus* from the Primary Breccia of the outer cave at Swartkrans (Figure 6), the upper iliac measurements, ***a, b, c, d*** and ***m*** had to be made from the reconstruction figure presented by Berge (1991). Figure 14 shows SK 3155b *Homo africanus* (Brain, Vrba and Robinson, 1974), and Figure 15, Sts 65, *Australopithecus africanus* from Sterkfontein Member 4 (Claxton et al., 2016). Neither preserved enough of the Robinson (1972) marker points to be included in the statistical analysis. Data for specimens which could be included in the quadratic discriminant analysis (QDA) are given in Appendix S2, along with statistical data from the QDA. Data for the three specimens which could not be retained in the QDA are given in the captions for the relevant Figures.

To evaluate the differences among the comparative sample for each variable mentioned in Table 1, Kruskal–Wallis tests were performed in SPSS 26. Coefficients of variation (CV) were calculated in Excel as the ratio of the standard deviation to the mean. Discriminant function analysis was used to investigate the multivariate morphology of the os coxae in the context of group membership, specifically to explore whether there was coherent grouping of the fossil hominin specimens included in our study. Some variables were missing in the hominin specimens (specifically ***e*** and ***f*** in MH2 and ***j*** and ***l*** in StW 431; see Appendix S2). Of the available variables, a forward stepwise approach identified ***m***, ***b**, **h**, **o*** and ***a*** for use in the model (*p* < 0.05 to enter). A Box's M test performed in SPSS 26 was highly significant (*p* < 0.001), indicating unequal covariances. Thus, quadratic discriminant function analysis was performed in JMP Pro 16.2 using the modern comparative sample as the training/validation set. Priors were set as proportionate to group size. Validation runs, using 10%, 20% and 50% of the sample, were performed. Alongside performing standard classification for the hominin specimens, the “consider new levels” function in JMP Pro 16.2, with a prior probability of 0.01 (roughly proportionate to the number of hominin specimens), was used to investigate whether the hominins might have a higher probability of belonging to an unspecified outlying group rather than to one of the comparative groups.

## 3 RESULTS AND PRELIMINARY DISCUSSION

### 3.1 Descriptive anatomy of StW 573

The dorsae of the left os coxa/innominate and the sacrum and coccyx are poorly preserved in StW 573 (Figure 2). The ventral surface (Figure 3) of the left os coxa is very thin and fragmentary, and is supported dorsally by cemented matrix. In Figure 1, we see the specimen prior to preparation. The ischiopubic ramus as shown in Figure 1 is prior to reconstruction and is attached to extraneous bony fragments, whereas Figures 7, 8, 9 show the cleaned and reconstructed ramus. The sacrum is present except for the right side of the coccyx, and the lumbar vertebrae are present but distorted. The condition of the left innominate, coccyx, sacrum and lumbar vertebrae, and the fragmented condition of the right innominate medial to the iliac pillar mean that we cannot discuss the obstetric pelvis here. However, enough exists of the sacrum and lower vertebral column to allow its virtual reconstruction, which is currently underway.

**FIGURE 16:**
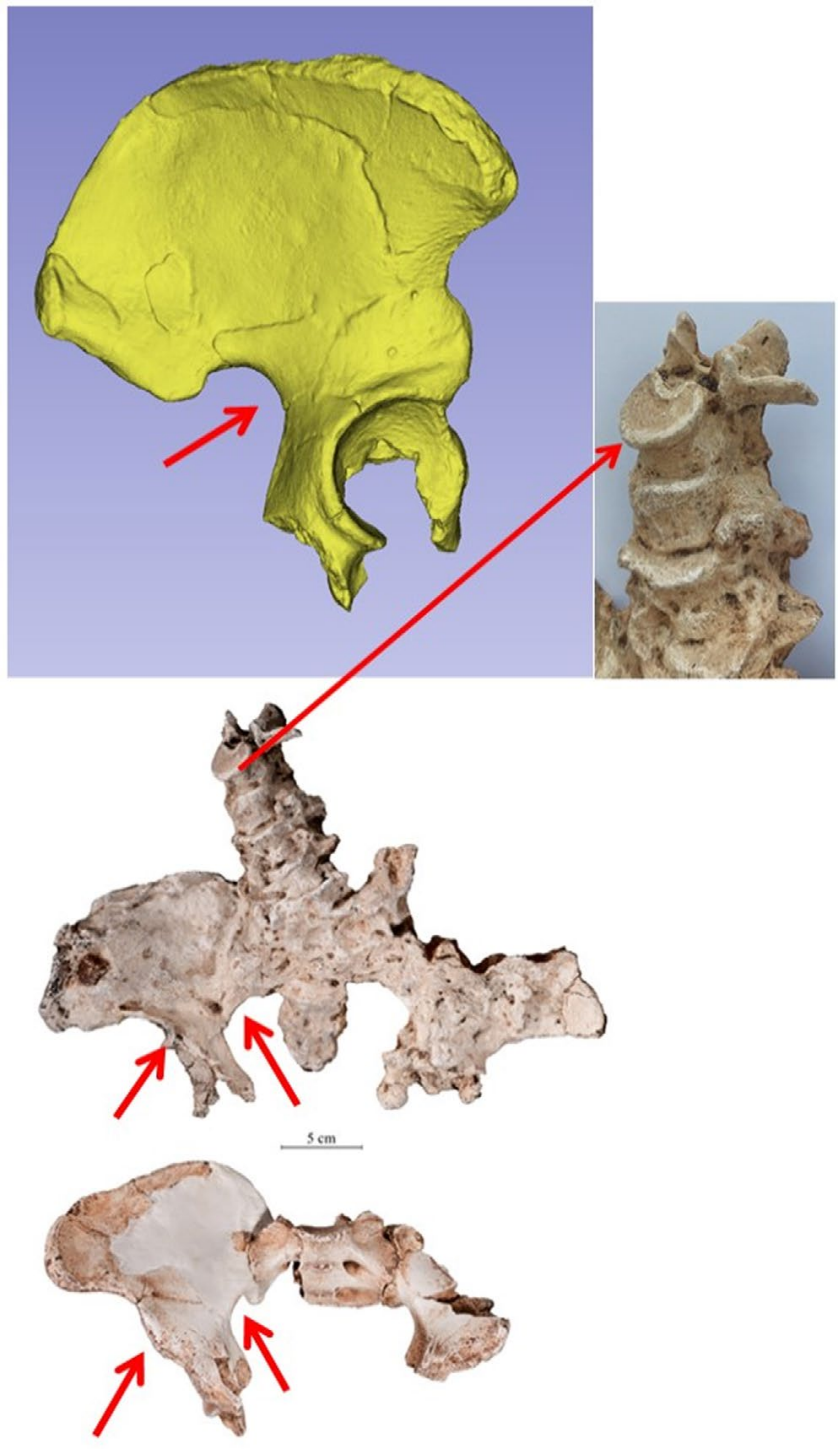
Casts of (top left and bottom) StW 431 and (middle) StW 573 to compare sciatic notch angles and anterior inferior iliac spine (short red arrows), and (top right) enlargement of the lumbar vertebral column of StW 573 (long red arrow) to show lipping of vertebral bodies.

Overall (see Figure 4b), the pelvis of StW 573 from Member 2 is remarkably similar in size and general appearance to that of StW 431 from Member 4 (Clarke, 2019a, 2019b), discussed in more detail below. StW 573 is female (Clarke & Kuman, 2019), whereas these authors have argued that as StW 431 has a more closed greater sciatic notch (see Figure 16), it is probably male. Anterior inferior iliac spine size may indicate sex better than the morphology of the sciatic notch (Gommery & Thackeray, 2006) but as the anterior inferior iliac spine is not well preserved in StW 573, no secure comparison based on this trait can be made with StW 431.

Given their similarities, it is likely that both StW 573 and StW 431 are best included together in *Australopithecus prometheus*, in contrast to the smaller Sts 14, which is referred to *Australopithecus africanus* (Häusler & Schmid, 1995). The iliac blade of StW 573 is thinner compared with that of StW 431, and thus it has been suggested that the two specimens are unlikely to be conspecific (Macho et al., 2020). However, the lipping of the vertebral bodies in StW 573 (see e.g. Figure 16) as well as closure of its cranial sutures (Clarke & Kuman, 2019), indicate that it is an old individual, whereas StW 431 is probably a relatively young individual (Gommery & Thackeray, 2006). (The lesions visible on L3 and L4 in StW 431 are likely due to early stages of brucellosis, according to D'Anastasio et al., 2009) On the basis of human lifetime changes in the bone of the hip (see e.g. Yates et al., 2007), it would be more likely that an old female would have a thinner ilium than a young male, so the difference in iliac blade robusticity in StW 573 and StW 431 may not be taxonomically informative.

The craniad third of the StW 573 iliac blade is apparently ventrally depressed, presumably by hard items in the matrix, but immediately dorsal to the anterior superior iliac spine, a line at the cranial margin of the depression may represent a linea glutea anterior. The flattening of the lateral iliac blade would have increased the apparent length of the iliac crest (and thus our measure of the distance between the anterior superior and posterior iliac spine, variable ***b***, see below) but the close size and shape match with StW 431 suggests the increase would be small. This may in future be clarified from the extensive virtual reconstruction currently underway. One of the most striking features of StW 573, shared with StW 431, is the extent of dorsad extension of the iliac blade and crest over the sacrum (see Figure 2). A pronounced dorsad extension of the blade and crest is also a feature of SK 50 (Figure 6). Haeusler (2002) states that dorsally, the iliac crest of StW 431 bears a posterior gluteal line indicating a substantial origin for gluteus maximus from the ilium, as in modern humans, where it acts to exert femoral extension in flexed hip postures, for example in humans when climbing the stairs (see, e.g. White et al., 2012). In StW 573, StW 431 and SK 50, this trait might be attributed to either, or both, tree climbing or bipedal walking in the hilly terrain that existed at the time of deposition. The prominent gluteal line also suggests a powerful gluteus medius, which would promote pelvic stability in standing and walking.

**FIGURE 17:**
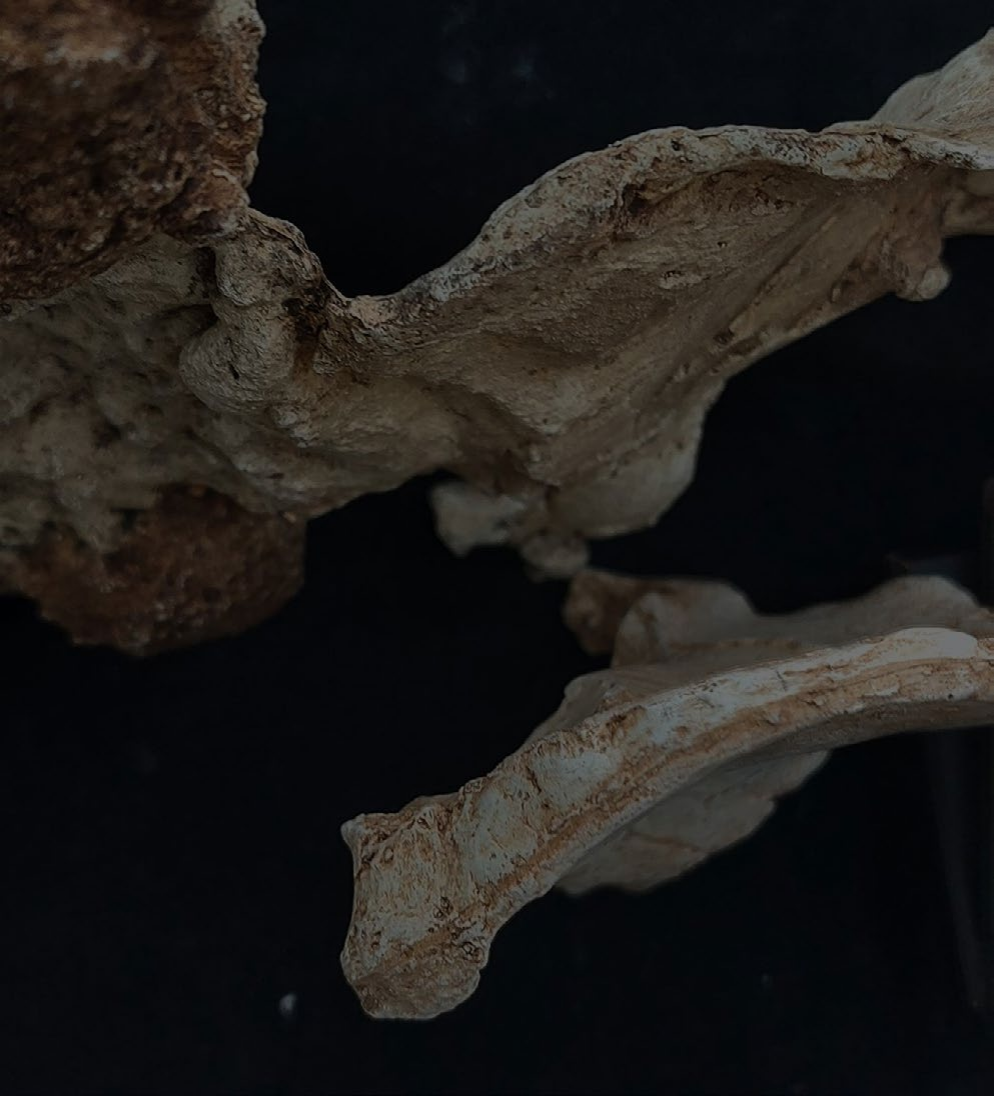
Cranial view of casts of (top) StW 573 bottom StW 431 to show the similarity of curvatures of the dorsal quarter of the iliac crests. Not to scale.

The dorsal quarter of the iliac crest of StW 573 bears the same sinuosity as that of the whole crest of StW 431 (Figure 17). Kibii et al. (2011), in reference to MH2, regard such sinuosity as a similarity to *Homo*. The dorsal quarter of the StW 573 crest bears labria externa and interna, as does much of the crest of StW 431, but while a linea intermedia is noted by Haeusler (2002) in StW 431, this marking is not apparent in StW 573. Following Haeusler (2002) the presence of these labria implies that the three muscles of the abdominal wall attached here, as in *Homo* but not *Pan*. More ventrally, the crest is narrower, and the extent of flattening/crushing (note, not distortion) of the ilium, which apparently occurred during burial, forbids confident statements about the existence of the labria, and the linea intermedia, in this region.

The posterior superior iliac spine (PSIS) of StW 573 is by no means as marked as that of StW 431. This may however simply be a reflection of advanced age and/or preservation. The anterior superior iliac spine (ASIS) is highly prominent in StW 573 and extends laterally beyond the acetabulum, as in StW 431 and SK 50, but significantly more so than in Sts 14, AL‐288‐1, *Ardipithecus ramidus* and WT‐15000. Following the anterior margin of the blade towards the acetabulum, the region of the anterior inferior iliac spine is damaged. However, both an iliopsoas groove and, immediately above the acetabulum, an iliopsoas ridge are clearly present, as in StW 431. Haeusler (2002) notes that this ridge may be partially present in Sts 14 but that it is completely absent in AL 288‐1. This might suggest locomotor distinctions between AL 288–1 and Sts 14, as well as distinctions from *Australopithecus prometheus*. It must always be borne in mind that the morphological distinctions we see are, in nearly all cases, represented in one or two individuals, not in populations. Thus, prima facie, it should be regarded as likely that such distinctions reflect individual differences in frequencies of use, or of context, of different limb excursion and/or muscle contraction patterns rather than wholescale differences in locomotor modes (such as erect versus bent‐hip, bent‐knee walking) and/or muscle attachment patterns.

The anterior margin of the acetabulum of StW 573 is missing, from immediately lateral to the iliopsoas groove, to just ventral to the caudal margin of the acetabulum, so the condition of the iliopectineal eminence, noted by Haeusler (2002) as salient in StW431, cannot be reported. The lunate surface, where present, is broad, as it appears to be in StW 431, but the acetabular fossa, the region of attachment of the ligament of the head of the femur, is narrow. The latter is missing in StW 431. The ventral margin of the acetabulum of StW 431, as much as remains ventral to its cranial extremity, has clearly been slightly dorsally flexed, resulting in some reduction of its likely maximum dorsoventral diameter.

**FIGURE 18:**
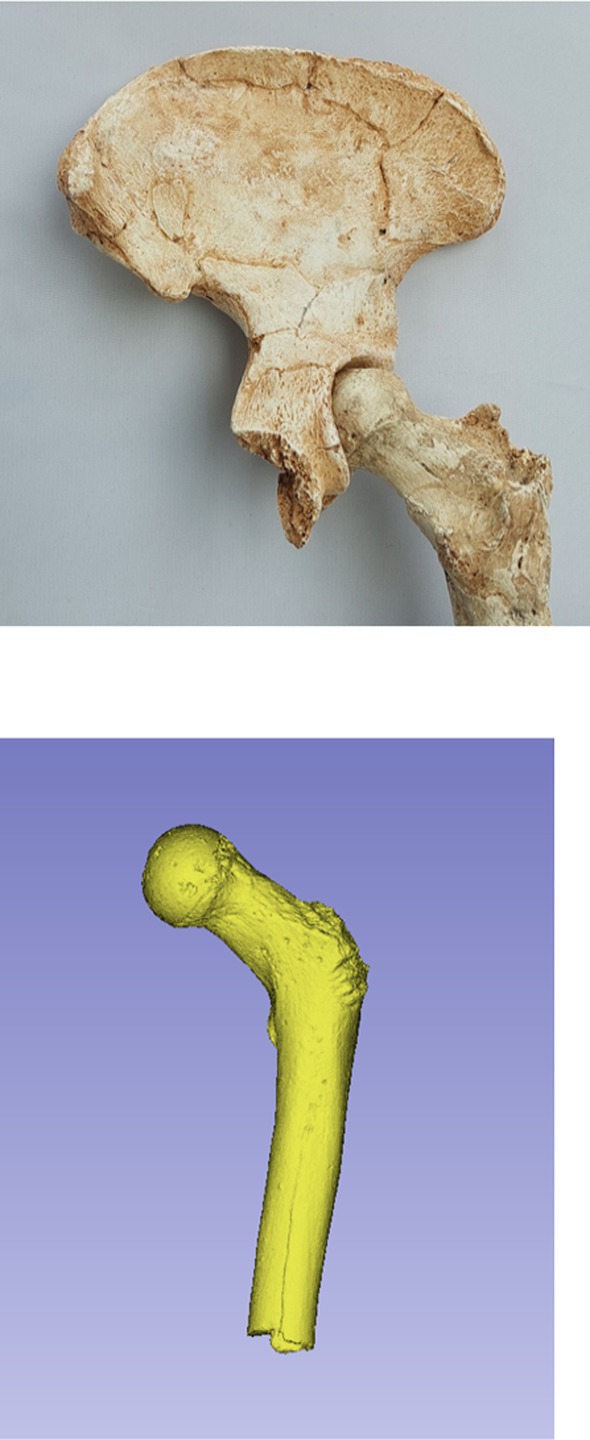
Above, The StW 573 proximal femur placed in the StW 431 acetabulum to show close size match: (both casts) StW 431 is the Kibii and Clarke (2003) reconstruction. Below, the StW 598 proximal femur from Jacovec Cavern (Pickering et al., 2021) Not to scale. Maximum diameter of femoral head of StW 598 31.8 mm.

From Figure 18 (top), we can see that at least qualitatively the StW 573 femoral head is a close match for the StW 431 acetabulum, and this finding is consistent with the short femoral neck of StW 573 compared with other Sterkfontein proximal femora assigned to *Australopithecus africanus*, such as that from Jacovec Cavern (StW 598; Partridge et al., 2003, Figure 18 bottom). It is worth noting that *Australopithecus afarensis* AL 288‐1 also has a short femoral neck. A future study should of course test such apparent similarities of shape/size using, for example the method of Hammond and Plavcan (2013).

**FIGURE 19:**
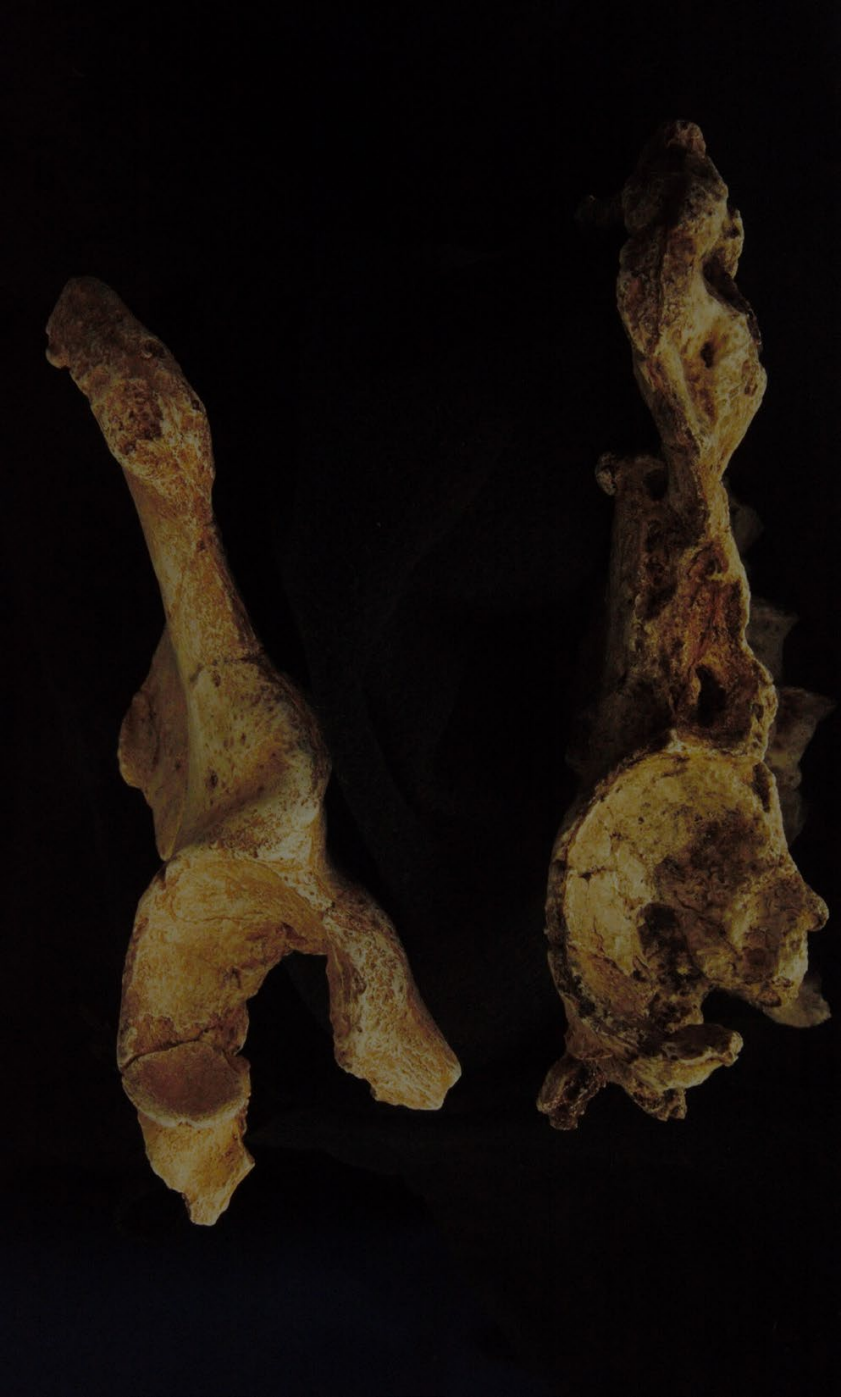
Casts of (left) StW 431 and (right) StW 573, from the lateral aspect to show the similarity of the acetabulum and iliac pillar region.

**FIGURE 20:**
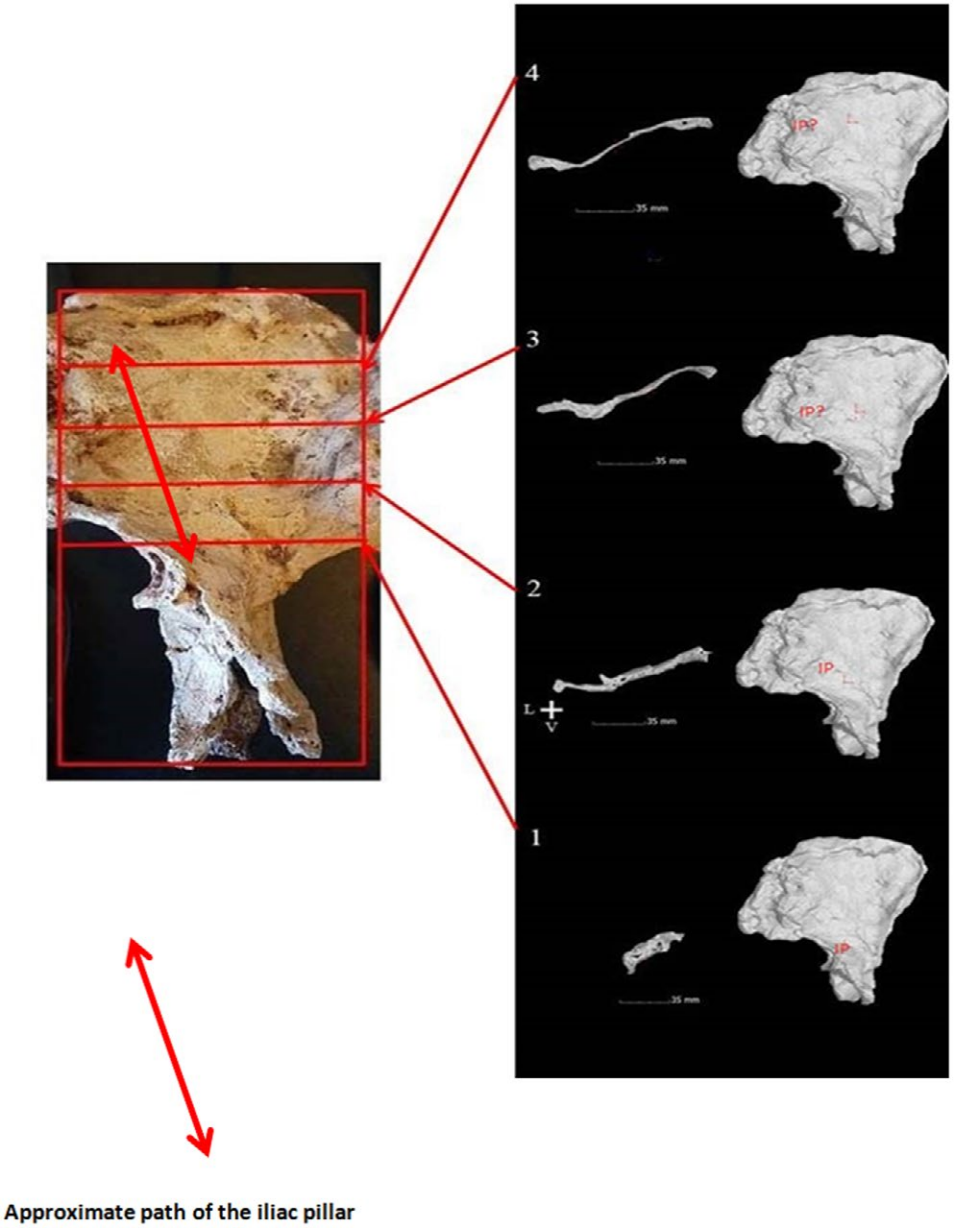
Right image: MicroCT sections through the iliac blade with, right, their position. Left image: The StW 573 innominate cast with iliac pillar position marked on it. (Images after Crompton et al. *Folia Primatologica* 2022, with publisher's permission.)

As shown from Figure 19, the morphology and size of the acetabulum and the iliac pillar region are very similar in StW 573 and StW 431. MicroCT sections through the pillar of StW 573 show that the pillar lacks strong boundary markings, slanting slightly towards the anterior superior iliac spine (Figure 20). Claxton et al. (2016) describe an isolated ilium from Sterkfontein Member 4, Sts 65 (and see Robinson, 1972), which they show has greater robusticity of the iliac pillar than have StW 431 and Sts 14, indicating greater vertical loading of the hip joint. The robusticity of the iliac pillar in StS 65 exceeds that observed in StW 573. This emphasises the considerable variation in locomotor mechanics among hominins at this time.

The ventral surface of the ilium preserves less detail than the dorsal surface, and little extra can be presented here until the virtual reconstruction currently under way is complete. The lateral one‐third has clearly been crushed, but the marginal outline is probably broadly much as in life, except for small notches missing at approximately 35% and 40% of its length medially from the anterior superior lilac spine. Caudally, an hollow exists dorsal to the medial acetabulum, reflecting the compression of the medial acetabular margin we have noted above.

The proximal ischium is preserved and unusually, although separate, a complete, originally highly fragmented but now restored right ischiopubic ramus (Figures 7, 8, 9) was discovered, including the pubic symphysis on both sides. Fragments of the margin of the obturator foramen exist but do not make a clean contact with the ramus. The ramus, like the innominate, does not retain any analytically usable muscle scars. Eyre et al. (2023) report a curious feature of the pubic symphysis in some australopiths, the ventral sulcus. They note that it may be a feature only of females, but do not identify a reason for that interpretation. They state that no ventral sulcus appears to be present in the pubic symphysis of StW 573, and indeed that the pubic symphysis is not visible. However, Figure 8 shows that, from the cranial perspective, the sulcus of StW 573 is a prominent morphological feature, bounded by prominent bony ridges, and is roughly ‘U'‐shaped. Figure 9, a dorsal perspective of the original StW 573 symphyseal fragment before reconstruction, shows, in addition to crystalline features, a very clear symphysis. Eyre et al. (2023) suggest that the australopith ventral sulcus could be a taphonomic feature arising from invertebrate burrowing. This seems unlikely in StW 573, a mummified (Clarke, 2019a, 2019b) skeleton untouched by any invertebrate interaction, which shows the feature very clearly. At present, the function (if any) of this marked anatomical feature remains unknown, which requires serious attention in future.

**FIGURE 21:**
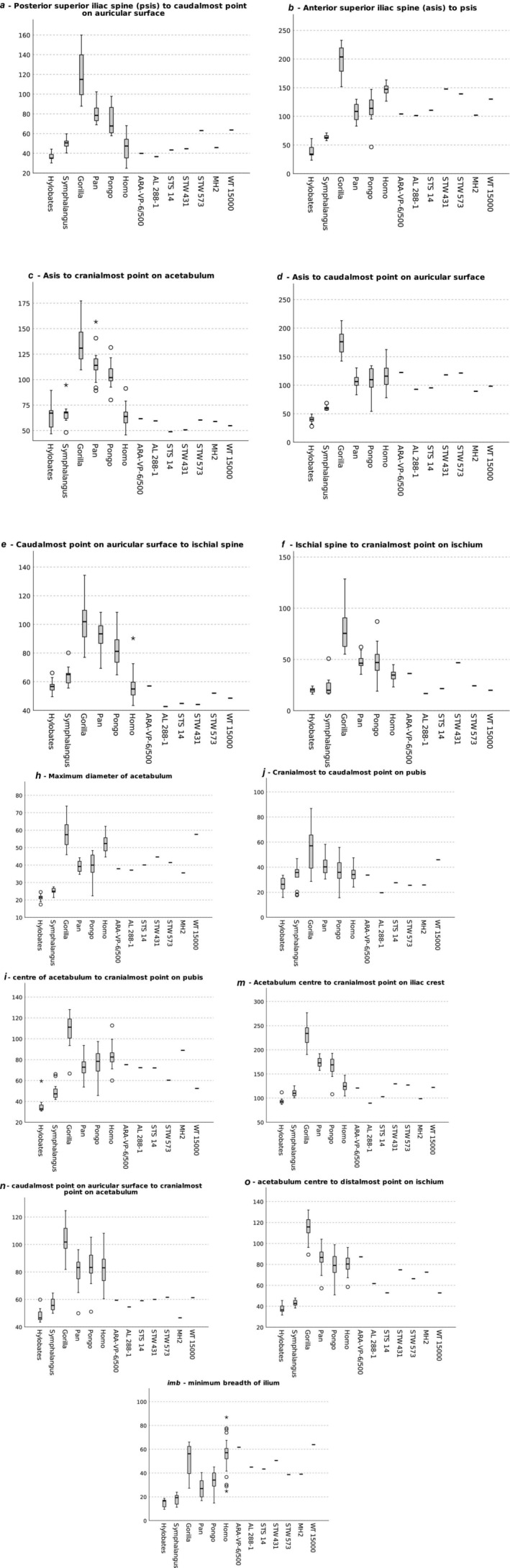
***a–f*** Independent Samples Kruskal–Wallis tests of variables ***a–f*** by taxon. In these plots, a circle indicates an individual value mildly outside the general range, a star a clear outlier. Measurements in mm. ***h‐imb*** Independent Samples Kruskal–Wallis tests of variables ***h–imb*** by taxon. In these plots, a circle indicates an individual value mildly outside the general range, a star a clear outlier. Measurements in mm.

**FIGURE 22:**
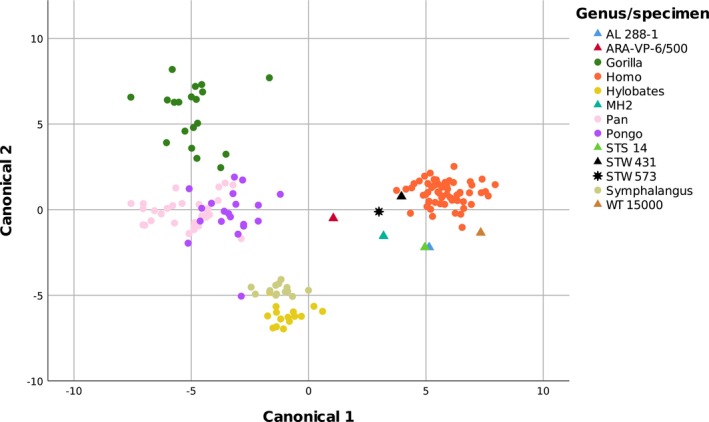
Plot of Canonical Variates 1 and 2. Note the distinct groupings of the extant comparative sample with the exception of *Pan* and *Pongo*.

**FIGURE 23:**
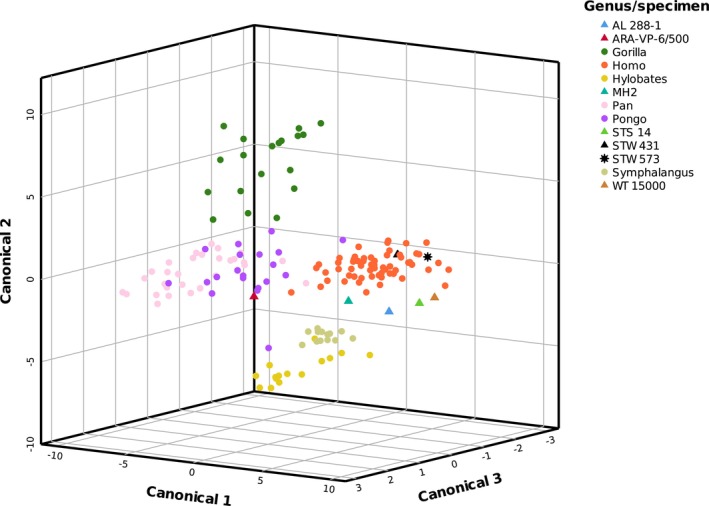
Plot of Canonical Variates 1, 2 and 3 showing *Adipithecus ramidus* falling within *Pongo*, StW 573 and StW 431 falling clearly within modern humans, and the other hominins occupying a more peripheral position close to modern humans.

### 3.2 Metric analysis

Metric data are summarised in Figure 21 ***a–f, h‐imb***. The full raw dataset, and details of the Quadratic Discriminant Analysis (Canonical Model, Canonical Details, Canonical Structure, and species statistics) are provided in Appendix S2. In Kruskal–Wallis tests, statistically significant differences (*p* < 0.001) among groups were noted for all 13 metric variables (listed in Table 1), although some post‐hoc pairwise comparisons, particularly between *Pan* and *Pongo*, were not significant. Table 3 gives the classification results for the QDA (based on the full training set), with the canonical details and structure of the model given in Appendix S2. Misclassification rates in the validation set across runs ranged from 6% to 17%, which indicated the relative robusticity of the model. Classification probabilities for the hominin specimens are listed in Table 4. In the standard QDA classification, all hominins other than *Ardipithecus ramidus* were assigned to the human group with very high probabilities. Inspection of the boxplots (Figure 21 ***a–f, h‐imb***) shows that in general, all the hominins fell within, or very near to, the range of variation evident in modern humans for the metric variables.

**TABLE 3:** Comparative sample sizes, percentages of specimens correctly identified by QDA and number of misclassifications.

Taxon	*N*	Specimens correctly classified	Misclassifications
Gorilla	19	18 (95%)	1 (5%) – orangutan
Human	59	59 (100%)	0
Gibbon	15	14 (93%)	1 (7%) – siamang
Chimpanzee and bonobo	29	28 (97%)	1 (3%) – orangutan
Orangutan	20	16 (80%)	4 (20%) – chimpanzee and bonobo
Siamang	13	13 (100%)	0
Total comparative sample	155	148 (95.5%)	7 (4.5%)

**TABLE 4:** Classification Results for Fossil Hominin specimens used in analysis.

Specimen	Taxon	Standard classification (probability)	Classification to unspecified outlying group (probability) by QDA “consider new levels” analysis in JMP 2023
ARA‐VP‐6/500	*Ardipithecus ramidus*	Orangutan (100%)	Unspecified (97%)
AL 288‐1	*Australopithecus afarensis*	Human (100%)	Unspecified (99%)
StW 573	*Australopithecus prometheus*	Human (100%)	Human (88%), unspecified (12%)
StW 431	*Australopithecus prometheus*	Human (100%)	Human (100%)
Sts 14	*Australopithecus africanus*	Human (100%)	Unspecified (94%)
MH2	*Australopithecus sediba*	Human (100%)	Unspecified (100%)
KNM‐WT 15000	*Homo ergaster*	Human (100%)	Unspecified (62%), human (38%)

The first three canonical functions of the QDA together contributed to over 99% of the discriminant model (Appendix S2), and Figures 22 and 23 show Canonical Function 1 versus 2 and Functions 1, 2 and 3, respectively. It is clear from these figures and the high classification success that there was excellent discrimination among the modern groups, although, reflecting the Kruskal–Wallis pairwise results, there was some misclassification between *Pan* and *Pongo* in particular. In the forward stepwise discriminant analysis, the variables ***m**, **b**, **h**, **o*** and ***a*** were selected as being the most informative discriminators of the modern groups (with ***m*** entered first and ***a*** last).

Variable ***m*** (the centre of acetabulum to the cranial most point on the iliac crest) describes the height of the iliac blade, and the shortened “bipedal” morphology is evident very clearly in Figure 22: despite the large body mass of humans, they are found towards the lower end of the extant comparative sample distribution (albeit above siamangs and gibbons). AL 288‐1 is at the extreme bottom end of the hominin distribution, reflecting its relatively small body mass alongside its shortened iliac blade. There is marked similarity in the reduced ilium height of StW 573 and StW 431. *Ardipithecus ramidus* lies very close to the human median, reflecting Lovejoy and colleagues’ (2009) observation (see in particular their Figure S10) that the height of the ilium in *Ardipithecus ramidus* is reduced in comparison to living nonhuman great apes. Lovejoy et al. (2009) note that consequence of reduction of posterior iliac height is that the lower lumbar vertebrae are not ‘entrapped’ as they are in living nonhuman great apes, facilitating lumbar lordosis and hence stabilisation of bipedal posture in the hominins. Lumbar lordosis is evident in StW 431 and by implication (given the state of preservation of the lowermost lumbar vertebrae) in StW 573 (see e.g. Figure 19).

Variable ***b*** (asis to psis) describes the anteroposterior width of the ilium. The absolutely larger size of the gorilla pelvis is evident, but the morphological difference of the human pelvis (the wider anteroposterior posterior dimension, creating the “basin” shape) is also clear (Figure 21). StW 573 and StW 431 have a wider ilium than the other hominins in the sample, including the larger‐bodied KNM‐WT 15000, for which the morphology may also be influenced by its juvenility. It may also be related to its gracile form, noted by Ruff and Walker (1993), with long legs and a narrow trunk, related primarily to exposed locomotion in a hot, arid environment. However, it may be that the Walker reconstruction requires revision: Torres‐Tamayo et al. (2021) alternatively propose, after predictive modelling, a broad pelvis with a laterally flared ilium, although currently it is unclear how that would affect the value of the traits we discuss here. AL 288‐1 shares a short femoral neck with StW 573, but diverges in iliac morphology, with a relatively anterioposteriorly narrow iliac blade, and it has been proposed (Wiseman, 2023, based on techniques developed by Brassey et al., 2018) that the lateral flaring of the iliac blades of AL 288‐1 offsets or circumvents the functional losses implied by the short femoral neck.

Maximum diameter of the acetabulum (variable ***h**)* is one notable exception to the pattern in which australopiths fell within modern human variation. Humans have relatively large joint surface areas in the lower limb, which likely reflects the demands of load bearing on two rather than four limbs (McHenry, 1992), and this is evident in the overlap between modern humans and the larger‐bodied gorillas in Figure 21 ***h***. The smaller acetabulum diameters in our australopith sample are consistent with previous observations (Ruff, 2010). KNM‐WT 15000, although juvenile, falls within the range for modern humans and is a similar value to the OH 28 *Homo erectus* acetabulum (as reported in Ruff, 2010), despite its juvenility and older geological age. It is possible that high hip‐joint forces, alongside body mass, are contributing to acetabular morphology in KNM‐WT 15000. The large acetabulum and long femur of KNM‐WT 15000 might also indicate long ranging distances compared with those of earlier hominins, and these traits may, perhaps, offset its relatively low iliac length.

Variable ***o*** (the centre of acetabulum to distalmost point on ischium) reflects both ischium length and acetabular diameter and thus extensor moment at the hip (see Aiello & Dean, 1990; Sigmon, 1974). A larger distance from the distal ischium to the centre of the acetabulum implies greater moment arms of the hamstrings and adductors about the hip joint. Based on hip extensor moment in the hamstrings modelled from iliac morphology and femur length in extant catarrhines (Kozma et al., 2018), living (non‐human) apes have a morphology which favours climbing ability but limits their range of hip extension, whereas humans reduce hip extensor moments but increase extension range and hence walking economy. The same study found that early hominins *Ardipithecus ramidus*, *Australopithecus afarensis* and *Australopithecus africanus* (Sts 14) balanced walking economy with climbing ability (Kozma et al., 2018). In our study, *Ardipithecus ramidus* falls within the human range in a high position. In contrast, Sts 14 and KNM‐WT 15000 all below it, indicating a short distance from the centre of the acetabulum to the distal ischium, which does not necessarily simply reflect differences in overall body size. The ischium is damaged in both StW 573 and STW 431, and so the estimated position was deliberately quite conservative, but these two specimens, alongside MH2, fall within the human range of variation. The pattern of diversity and its functional correlates in our sample are thus unclear and future work investigating hamstring extensor moments versus extension range would be valuable.

Following Haeusler (2002) variable ***a*** (posterior superior iliac spine to caudalmost point on auricular surface) is a measure of the breadth of the iliac origin of gluteus maximus, and partly of that of gluteus medius. KNM‐WT 15000 and STW 573 both lie at the upper end of the human distribution, whereas the other fossil hominins lie around the median and first interquartile range (Figure 21a). This variable shows marked variation within the hominins, with a CV very similar to that in humans (Table 5). In early hominins, several of the other variables (***b, f, h, j, l, m, o)*** exceed the variation evident in the modern human sample (Table 5). In some variables (***b, f, m***), australopith variation was even more marked than in the fossil hominin sample as a whole (Table 5). The effects of this variation were reflected in the QDA “consider new levels” analysis (see Table 4), under “Classification to group (probability)” which split the early hominin sample between the human group (StW 431, StW 573, possibly KNM‐WT 15000) and one or more unspecified groups (*Ardipithecus ramidus*, AL 288‐1, Sts 14, MH4). StW 431 and StW 573 were assigned to the human category, but the other hominins were assigned to an unspecified group, but with a relatively low probability for KNM‐WT 15000, which also had a reasonable probability of classification to the human group (Table 4). Given the clear indications of habitual, human‐like bipedalism in its skeleton as a whole (Walker & Leakey, 1993), we cannot rule out that the uncertain classification in KNM‐WT 15000 might be because of its juvenility. In Figure 23, StW 431 and StW 573 group with *Homo* and away from the other hominin specimens, which otherwise cluster quite closely together at the edge of the human group, with the exception of *Ardipithecus ramidus*, which lies nearer *Pongo*.

**TABLE 5:** Coefficients of variation for the samples.

Variable	Coefficient of variation (CV)							
	Gibbon	Siamang	Gorilla	Chimpanzee/bonobo	Orangutan	Human	All fossil hominins	Australopiths^a^
***a***	0.11	0.10	0.18	0.11	0.20	0.26	0.22	0.21
***b***	0.29	0.06	0.13	0.14	0.19	0.06	0.16	0.18
***c***	0.21	0.15	0.13	0.12	0.11	0.13	0.09	0.10
***d***	0.14	0.06	0.12	0.11	0.19	0.15	0.14	0.15
***e***	0.07	0.10	0.13	0.10	0.14	0.14	0.11	0.09
***f***	0.12	0.40	0.24	0.12	0.29	0.16	0.42	0.49
***h***	0.08	0.07	0.14	0.08	0.16	0.08	0.18	0.09
***j***	0.22	0.27	0.31	0.19	0.28	0.15	0.31	0.14
***l***	0.20	0.16	0.13	0.12	0.18	0.10	0.18	0.16
***m***	0.07	0.07	0.10	0.06	0.11	0.08	0.14	0.16
***n***	0.09	0.08	0.11	0.12	0.14	0.13	0.09	0.11
***o***	0.10	0.07	0.10	0.11	0.15	0.09	0.19	0.13
***imb***	0.22	0.23	0.27	0.28	0.22	0.19	0.21	0.11

^a^ AL 288‐1, StW 573, StW 431, Sts 14, MH2.

## 4 DISCUSSION AND CONCLUSION

Other than in the heuristic *Ardipithecus ramidus* model, ossa coxae morphology in our hominin sample showed strong affinity to that of humans, reflecting the profound adaptive changes in the pelvis because of bipedalism. Whether the traits that group fossil hominins with modern humans represent synapomorphies (as suggested for the case of *Australopithecus sediba* and *Homo* by Kibii et al., 2011) requires further investigation, especially given the variation evident in the fossils. This notwithstanding, the morphological similarities among most of the hominin sample underline the notion of a common *Australopithecus* “bauplan”, with at least some variation relating to realised niche (Crompton et al., 2022). The exception, *Ardipithecus ramidus*, which was assigned to the orangutan group, has been described as representing a “transition from arboreal life to habitual terrestrial bipedality” (Lovejoy et al., 2009: p. 48). Its grouping is particularly interesting in the light of Thorpe et al. (2009) observations on arboreal bipedality in *Pongo*. The *Ardipithecus ramidus* result also aligns with a previous conclusion based on ARA‐VP‐6/500 that pelvic morphologies in *Pan* and *Gorilla* are derived (Lovejoy et al., 2009), although there is considerable debate about *Ardipithecus ramidus* morphology and affinities (see for example Prang, 2019).

Craniodental differences noted by Clarke (1998, 2008, 2013) and Clarke and Kuman (2019) in the Sterkfontein australopiths suggest the presence of more than one hominin species at Sterkfontein, notwithstanding other studies that are ambivalent about this (Grine, 2019). The indisputable association between the StW 573 skull and pelvis allows us to have confidence in describing and quantifying the innominate morphology of *Australopithecus prometheus* and thus investigating its affinities with other fossil ossa coxae. StW 431 has a qualitatively very similar os coxae morphology to StW 573 and the two specimens appear to have similarities in several metric variables, including an anteroposteriorly wide ilium. In contrast to the other hominins, *Australopithecus prometheus* StW 573 and StW 431 were assigned in the “consider new levels” analysis to the human rather than the uncategorised group, occupying a canonical space away from the other hominins, including Sts 14. It is unlikely that the multivariate morphology that groups StW 573 and StW 431 firmly within the range of modern humans reflects a set of derived traits that imply a closer evolutionary relationship between *Australopithecus prometheus* and modern humans compared with the other hominins in the sample. However, it is likely to reflect a close relationship between StW 573 and StW 431. Taxonomically‐meaningful differences in morphology between the first two sacral vertebrae of Sts 14 and StW 431 have been observed (Fornai et al., 2021), and our findings thus add to the postcranial evidence that strongly supports the concept of two contemporaneous *Australopithecus* species at Sterkfontein. Thus, StW 431 belongs to *Australopithecus prometheus*, alongside StW 573, whereas the morphologically different Sts 14 represents *Australopithecus africanus*. It is likely that the two Sterkfontein species differ ecomorphologically and that the differences we and others observe reflect the interplay between realised niche and taxonomy.

This interplay may result in distinctions in morphology in the relatively simple ossa coxae of early hominins which would serve, but do not necessarily imply, distinctions in locomotor behaviour. Some of the morphological variation we observe in ossa coxae (which for several traits exceeds variation in most of the comparative sample by a considerable margin) may reflect lifetime behavioural (i.e. plastic) differences between individuals. Other differences, such as those observed between *Ardithecus ramidus* and other early hominins, and (since they come from the same site and overlapping time‐frames) those between StW 573 and StW 431, on the one hand, and Sts 14 on the other, are probably taxonomically meaningful. We cannot rule out that the classification of *Ardipithecus ramidus* with *Pongo* rather than with the other hominins and humans indicates that there may be multiple forms of bipedality represented in our os coxae sample. This conclusion is reinforced by the “consider new levels” analysis, which split the hominin sample between the human group (StW 431, StW 573, possibly KNM‐WT 15000) and one or more unspecified groups (*Ardipithecus ramidus*, AL 288‐1, Sts 14, MH4). Although this function in JMP is relatively new, in allowing specific identification of outliers to existing groups it provides an interesting extension to traditional discriminant analysis, which is otherwise designed to assign unknown specimens to existing categories.

The variation we identify is particularly interesting in that it provides another line of evidence for diversity in bipedal behaviour. Most previous evidence for this has been drawn from the foot and footprints (e.g. Haile‐Selassie et al., 2012; McNutt et al., 2021), interpretation of which is made more complex by evidence of very high variability in modern human foot pressure (e.g. McClymont et al., 2021) and by the broad scientific acceptance of the theory of neurobiological degeneracy, which predicts that in complex multi‐element appendages, the same external forces can be generated by many, differently shaped, hard and soft tissue interactions. Although redundancy in joint function is still likely in the pelvis, its simpler structure plus its lack of direct interaction with the substrate makes interpretation of its form‐function correlates more robust. Even so, the os coxae variation observed here is not unequivocal evidence for multiple forms of bipedality, and we stress the need for caution in drawing conclusions about the patterns and processes implicated in the evolution of bipedalism(s) until multiple analyses have been performed using different variable‐sets and analytical approaches. What we do argue with confidence, however, is that the structural variation in hominin os coxae seen in this sample promotes ecological robusticity, as a range of different locomotor outputs can be produced, in a range of ecological contexts. Furthermore, degeneracy is acting at the genomic level, sustaining variation to favour future generations in changed environments. Thus, the ecomorphological variation in os coxae morphology in our fossil ancestors is likely to have sustained our evolvability.

## AUTHOR CONTRIBUTIONS

This paper forms part of the documentation of StW 573 by the core research team led by R.J. Clarke, and all authors contributed ideas, concepts and background knowledge to the paper. In particular, Crompton collected the data, conceived and wrote the manuscript; Elton performed statistical analysis; Sellers created computer models; Pickering contributed data; Hirasaki and Scott obtained surface scans; Clarke and Kuman provided background, images, advice and editing; and McClymont virtually segmented the original innominate scans, and contributed key theoretical concepts.

## FUNDING INFORMATION

Data shared by Carol Ward was gathered under support from Wenner ‐Gren Foundation and L.S.B. Leakey Foundation to herself and National Science Foundation NSF BCS 0716244 and NSF BCS 0647557 to J. Michael Plavcan.

## DEDICATION

RHC dedicates this paper to Christine Berge, whose outstanding career was sadly cut short by illness. JMcC dedicates this paper to Douglas Duncan McClymont, a life‐changing teacher, beloved father, and paradigm shifting sports biomechanicist.

## Supporting information


Appendix S1.

Appendix S2.

## Data Availability

The fossil hominid scans used in this study are available on request from Transvaal Museum and The University of the Witwatersrand, and from Morphosource. Restrictions apply to the availability of the Transvaal Museum and University of the Witwatersrand specimens. Those from Morphosource required the agreement of the person who uploaded them. Measurements made by the first author are provided in Appendix S2.
