# Supplementary material for: Bipedalism or bipedalisms: The os coxae of StW 573
Source: J Anat. 2024 Jul 22;248(2):163–83. doi: 10.1111/joa.14106 (PMC12779419; doi:10.1111/joa.14106)
Supplement: Supplementary file 1 — Appendix S1. [file JOA-248-163-s002.docx]

**Supplementary Material 1: Subject Selection**

Most of the living hominoids are those used by Carol Ward in her publications on the os coxae, with the addition of some extra scans from the Kyoto Primate Centre. In the first place we needed to test the hypothesis that there are at least two hominin species present at Sterkfontein: the StW 431 scan was taken from the Kibii and Clarke (2003) reconstruction, being that derived from the most complete fossil data. The Sts 14 pelvis reconstruction is as reconstructed by Berge and Gouleras (2010) as Clarke and RHC have first-hand knowledge of Berge’s reasoning. We would have wished to be able to include SK 50 *Paranthropus* given its role in the considerations of Robinson (1972) but too many measures could not be assessed. Haeusler’s (2002) important study of the pelvis compares the South African *Australopithecus* pelves with that of AL 288-1 *Australopithecus afarensis* as reconstructed by Tague and Lovejoy (1986) so that reconstruction was included. The earliest evidence of the hominin pelvis is that of *Ardipithecus ramidus* as reconstructed by Lovejoy et al. (2009) so that needed to be included, and we included the Walker and Ruff (1993) KNM WT 15000 pelvic reconstruction to represent early *Homo* as a comparator. It was not possible to include *Homo naledi* (see eg. Van Sickle et al. 2018) as it is highly fragmentary and the Robinson (1972) marker set we use could thus not have been applied with any confidence. We did however include *Australopithecus sediba* MH2 in the Kibii et al. (2011) reconstruction as these authors report close similarities to Homo. We were unable to measure sufficient Robinson (1972) variables on another likely *Paranthropus*, SK 3155 b (Brain, Vrba and Robinson 1972) nor on *Australopithecus africanus* Sts 65 (Brain, Vrba and Robinson 1972 and see Claxton et al. 2016) to include them in the analysis but for convenience, present what data we could gather with their Figures.
